# Mechanistic Insights into the FOXM1/BUB1 axis-Mediated Oncogenic Signaling in Hepatocellular Carcinoma

**DOI:** 10.7150/ijbs.125454

**Published:** 2026-02-26

**Authors:** Shuping Wang, Yudong Mao, Tingyu Zeng, Tao Yong, Yu An, Jipin Li, Yuan Wang, Xiaojun Yang, Quanlin Guan

**Affiliations:** 1 Key Laboratory of Preclinical Study for New Drugs of Gansu Province, Institute of Biochemistry and Molecular Biology, School of Basic Medical Sciences, Lanzhou University, Lanzhou 730000, PR China.; 2 The First School of Clinical Medicine, Lanzhou University, Lanzhou 730000, PR China.; 3 The Second Hospital & Clinical Medical School, Lanzhou University, Lanzhou 730000, China.; 4 Department of Hepatobiliary Surgery, Gansu Provincial Hospital, Lanzhou 730000, China.

**Keywords:** hepatoma carcinoma, FOXM1, BUB1, DNA repair, stemness

## Abstract

The development of novel therapeutic strategies for advanced and metastatic hepatocellular carcinoma (HCC) remains an urgent clinical need. Despite suboptimal efficacy, the breakthrough of tyrosine kinase inhibitors in HCC treatment therapy underscores the advantage of targeted therapy. Therefore, innovative targeted therapies are urgently needed to enhance treatment efficacy, decrease recurrence rates, and improve patient survival outcomes. The forkhead box M1 (FOXM1) transcription factor serves as a master regulator of oncogenic signaling networks that drive cancer progression. Our study identified budding uninhibited by benzimidazoles 1 (BUB1) as a crucial downstream effector of FOXM1, with demonstrated direct protein-protein interaction. Moreover, FOXM1 directly bound to the GTAAACC motif at the -293 bp region of the BUB1 promoter and activated its transcription, thereby driving HCC cell proliferation. Mechanism studies have shown that the FOXM1/BUB1 axis regulated multiple oncogenic processes in HCC, including cell proliferation, DNA repair, G2/M cell cycle transition, stemness, invasion, and migration. Knockdown of BUB1 significantly sensitized HCC cells and xenograft tumors to the FOXM1 inhibitor FDI-6. Furthermore, combined pharmacological inhibition of FOXM1 (FDI-6, RCM-1, thiostrepton) and BUB1 (BAY-1816032) synergistically inhibited the proliferation of HCC cells and xenograft tumors. These findings establish FOXM1-mediated BUB1 upregulation as a key driver of HCC malignancy. Targeting the FOXM1/BUB1 axis represents a promising therapeutic strategy for the treatment of advanced and metastatic HCC, offering new opportunities for HCC therapy.

## Introduction

The development of novel therapeutic strategies against advanced and metastatic malignancies remains an urgent global health priority, as these conditions continue to account for the majority of cancer-related mortality worldwide 1,2. Targeted therapy has become a promising paradigm in current cancer treatment 3-6. However, existing targeted agents face significant clinical limitations, particularly the emergence of therapeutic resistance, underscoring the critical need to identify novel molecular targets and develop more effective treatment modalities for refractory cancers 3,7. As master regulators of oncogenic signaling networks, cancer-associated transcription factors constitute pivotal therapeutic targets 8. Among these, forkhead box M1 (FOXM1) has been established as a central molecular orchestrator of multiple cancer hallmarks, including cell cycle regulation, proliferative signaling, metastatic progression, tumor recurrence, and stemness maintenance 9-11. Importantly, FOXM1 has been demonstrated to functionally modulate therapeutic responses across diverse treatment modalities, including targeted therapies, conventional chemotherapies, and immune checkpoint inhibitors 11,12. Therapeutic targeting of the oncogenic transcription factor FOXM1 represents a promising strategy to enhance clinical outcomes in multiple cancer types and circumvent multidrug resistance. Several FOXM1-targeted agents are currently under active investigation, including small-molecule inhibitors, proteolysis-targeting chimeras (PROTACs), peptide-based inhibitors, and targeted protein degraders 13-16. The cancer-specific functional profile of FOXM1 strongly supports its potential as an effective therapeutic target in oncology.

Hepatocellular carcinoma (HCC) ranks as the sixth most commonly diagnosed malignancy and fourth leading cause of cancer-related deaths worldwide, representing an aggressive hepatocyte-derived cancer with persistently poor prognosis and projected increasing mortality rates over the coming decade 17. Established etiological factors include chronic hepatitis B/C viral infections, non-alcoholic fatty liver disease (NAFLD), and alcohol-associated liver disease, all of which drive progressive hepatic fibrosis, cirrhosis, and eventual carcinogenesis 17,18. Clinical management remains particularly challenging due to intrinsic therapeutic resistance and high recurrence rates 18,19. Early-stage HCC is typically managed through locoregional interventions including surgical resection, radiofrequency ablation, transarterial chemoembolization (TACE), or liver transplantation 18,19. However, the majority of HCC cases are diagnosed at advanced stages when tumors become unresectable and conventional locoregional therapies demonstrate limited efficacy. In these cases, systemic treatment modalities encompassing chemotherapy, molecular targeted therapy, immunotherapy, and oncolytic virotherapy are employed to improve clinical outcomes 18-22. Notably, tyrosine kinase inhibitor-based regimens remain the therapeutic mainstay for advanced HCC, despite demonstrating suboptimal efficacy 23. The persistently high mortality rate highlights the urgent need for novel therapeutic targets and innovative treatment strategies to enhance therapeutic efficacy, reduce recurrence rates, and substantially prolong survival in patients with advanced HCC.

As a key transcription factor, FOXM1 exerts its oncogenic functions primarily through modulation of downstream target gene expression 9. Our prior research established that the FOXM1 inhibitor FDI-6 suppresses malignant proliferation in pancreatic cancer through downregulation of budding uninhibited by benzimidazoles 1 (BUB1) expression 24. BUB1, an evolutionarily conserved serine/threonine kinase, serves as a critical component of the spindle assembly checkpoint, with essential functions in chromosome alignment and mitotic progression regulation 25. Increasing evidence establishes BUB1 as an oncogenic driver in various types of cancer 25,26. Notably, BUB1 inhibition has been proven to be able to overcome the resistance of lung cancer to radiotherapy and chemoradiotherapy 27. In pancreatic cancer, BUB1 promotes gemcitabine resistance through ferroptosis suppression 28. Furthermore, BUB1 regulates DNA repair via the non-homologous end joining pathway, maintains cancer stem cell properties, and enhances therapeutic resistance in breast cancer patients receiving chemotherapy and radiotherapy 29,30. Collectively, BUB1 regulates cancer progression in multiple aspects, including DNA repair, stemness maintenance, metastatic dissemination, mitotic regulation, and ferroptosis modulation, while promoting radio-resistance and chemoresistance, making it a promising therapeutic target. Several small molecule inhibitors of BUB1 have been developed 31. However, monotherapies face significant limitations including restricted clinical applicability, rapid resistance emergence, and poor efficacy 3,7. Although the potential biomarkers for predicting the efficacy of the FOXM1/BUB1 axis are still unknown. Investigation of the FOXM1/BUB1 axis and development of multi-target intervention strategies may provide more effective therapeutic options for HCC and other malignancies. This approach holds promise to circumvent the constraints of single-target therapies while simultaneously enhancing treatment outcomes and patient survival.

In this study, we systematically investigated the regulatory relationship between FOXM1 and BUB1, and elucidated its mechanistic contributions to HCC progression through comprehensive analysis of DNA repair, cell cycle progression, stemness maintenance, and metastatic potential. Our investigations revealed significant overexpression of FOXM1 and BUB1 in both HCC tissues and cell lines, with strong correlation to adverse clinical outcomes. Transcriptomic profiling via RNA sequencing (RNA-seq) demonstrated FOXM1-mediated promotion of HCC malignancy by enhancing DNA repair capacity, accelerating cell cycle progression, maintaining stemness properties, and facilitating migratory and invasive potential. Mechanistically, we established BUB1 as a direct transcriptional target of FOXM1, with chromatin immunoprecipitation Q-PCR (ChIP-qPCR) assays confirming FOXM1 binding to the GTAAACC motif at the -293 bp region of the BUB1 promoter. Functionally similar to FOXM1, BUB1 promotes HCC proliferation, DNA repair, cell cycle progression, stemness acquisition, and metastatic potential. Importantly, knockdown of BUB1 significantly enhanced HCC cell sensitivity to the FOXM1 inhibitor FDI-6. Furthermore, combinatorial treatment with the BUB1 inhibitor BAY-1816032 (BAY) and FOXM1 inhibitor (FDI-6, RCM-1, or thiostrepton (TST)) synergistically suppresses HCC proliferation, DNA repair efficiency, cell cycle progression, stemness maintenance, and metastatic capacity. These findings provide transformative insights for HCC therapeutics, highlighting the potential of a coordinated approach targeting the FOXM1/BUB1 axis. This strategy represents a novel multi-modal therapeutic avenue for the management of advanced and metastatic HCC, particularly in tumors exhibiting high expression levels of FOXM1 and BUB1.

## Materials and Methods

### Bioinformatics analysis

The differential expression of genes in liver hepatocellular carcinoma (LIHC) tissues and adjacent normal tissues from the cancer genome atlas (TCGA) database was analyzed using the gene expression profiling interactive analysis (GEPIA) online tool (http://gepia2.cancer-pku.cn/#index). P-values were calculated using the Kruskal-Wallis one-way ANOVA test, with significance thresholds set at 0.05 for the P-value and 2.0 for fold change. The differential expression of FOXM1 in HCC tissues and adjacent normal tissues from the human protein atlas was analyzed using the online tool (https://www.proteinatlas.org/). The relationship between gene expression and the survival of patients with HCC was evaluated using the Kaplan-Meier plotter. The samples were divided into high-expression group and low-expression group based on the median expression level. The 95% confidence interval, log-rank hazard ratio (HR), and P-value for overall survival (OS) and disease-free survival (DFS) were evaluated. Original clinical data containing RNA-seq information from 424 HCC cases were sourced from the TCGA database (https://portal.gdc.com). Prognostic correlations were analyzed using receiver operating characteristic (ROC) curves. ROC curves and area under the curve (AUC) values were generated using the R package pROC and visualized via ggplot2. AUC value ranging from 0.5 to 0.7 indicates an average model performance, a value between 0.7 and 0.9 indicates strong performance, and a value greater than 0.9 indicates extremely strong performance. All statistical analyses and visualizations were conducted in R version 3.6.3. Expression correlations between key genes were analyzed using the GEPIA2 online tool. FOXM1 and BUB1 mRNA expression distribution in HCC cell lines was derived from the cancer cell line encyclopedia (CCLE) dataset (https://portals.broadinstitute.org/ccle). Analyses were performed using R v4.0.3 and ggplot2 (v3.3.3). FOXM1 ChIP-seq data were obtained from the cistrome data browser (http://cistrome.org/db/#/). Top50 targets in the PPI network of FOXM1 and BUB1 were analyzed using the STRING on line software (https://cn.string-db.org/). The common targets in DEGs of FOXM1 knockdown RNA-seq and in the PPI network of FOXM1 were predicted using the BioVenn online software. The biological characteristics and functions of common genes were analyzed by GO and KEGG pathway enrichment using the Omicsmart online platform. We employed the Oncogenic Cancer Lineage as a Regression algorithm developed by Malta et al. to evaluate the relationship between gene expression and tumor stemness. STAR-counts data and corresponding clinical information for LIHC were downloaded from the TCGA database (https://portal.gdc.cancer.gov). We then extracted data in TPM format and performed normalization using the log_2_(TPM+1) transformation. After retaining samples that included both RNA-seq data and clinical information, we ultimately selected 371 samples for further analysis. Gene expression profiles were processed using Spearman correlation analysis, with subsequent linear transformation to standardize stemness indices within the [0,1] range. All the above analysis methods and R package were implemented by R foundation for statistical computing (2020) version 4.0.3. P-value < 0.05 was considered statistically significant.

### Lentivirus Infection

Lentiviral recombination vectors encoding human FOXM1 complete DNA (FOXM1 cDNA) (pGV341-cFOXM1), human BUB1 complete DNA (BUB1 cDNA) (pGV341-cBUB1), empty vector control (pGV341-cNC), lentiviral shRNA vectors targeting FOXM1 (pGV112-shFOXM1), BUB1 (pGV112-shBUB1), and scrambled control (pGV112-shNC) were constructed and purchased from Genechem Co. Ltd. (Shanghai, China). HUH7 and HepG2 cells were infected with FOXM1 cDNA, BUB1 cDNA, NC cDNA, FOXM1 shRNA, BUB1 shRNA, and NC shRNA lentiviral vectors using HitransG P transfection reagent according to the protocol of manufacturer. After 2-3 days of lentiviral infection, the virus-containing medium was removed. Transfected cells were cultured for 2-3 days, followed by 24 h treatment with 2.0 μg/mL puromycin to select positively infected cells. For rescue experiments, cells were first infected with one lentiviral vector for 3 days, after which the virus-containing medium was removed. They were then infected with a second vector for 3 days, and the virus-containing medium was again removed. Cells transfected with both vectors were cultured for 2-3 days before subsequent experiments. All transfected cells were validated by Q-PCR and Western blot. The target sequences were as follows: FOXM1 shRNA, 5'-CAGGCTGCACTATCAACAATA -3'; BUB1 shRNA, 5'-ACCAGTGAGTTCCTATCCAAA-3'; and NC shRNA, 5'-CATTCTCCGAACGTGTCACGT-3' 32.

### Cell culture

The human HCC cell lines HUH-7 (SCSP-526) and HepG2 (SCSP-510) were purchased from cell resources center of Shanghai academy of life sciences (Shanghai, China), the test results of bacteria, mycoplasma and fungi were all negative. HUH-7 cells and HepG2 cells were cultured in Dulbecco's Modified Eagle Medium (KGL1206-500, KeyGEN Biotech, Nanjing, Jiangsu, China) with 10% fetal bovine serum (FBS). All cells were incubated at 37 °C in a 5% CO2 atmosphere 24,32.

### RNA-seq and data processing of DEGs

HUH7 cells were transfected with FOXM1 shRNA or NC shRNA for 3 days. Following treatment, cells were harvested, and total RNA was extracted using TRIzol reagent (Vazyme, Nanjing, China) according to the instruction of manufacturer. A total of 600 ng RNA was used to construct libraries with the NEBNext Ultra RNA Library Prep Kit for Illumina. RNA quantity and quality were evaluated using an Agilent 2100 Bioanalyzer. The RNA libraries were sequenced on an Illumina HiSeq™ 2500/4000 platform by Gene Denovo Biotechnology Co., Ltd. (Guangzhou, Guangdong, China). DEGs between the NC KD group and FOXM1 KD group were identified using thresholds of |log_2_FC| > 1.0 and adjusted P < 0.05. DEGs with log_2_FC < -1.0 were classified as downregulated, while those with log_2_FC > 1.0 were classified as upregulated. The raw RNA-seq data have been deposited in the SRA database under accession number PRJNA1272254 24,32.

### GO and KEGG pathway enrichment analysis

The biological characteristics of the differentially expressed genes (DEGs) were determined through Gene ontology (GO) enrichment analysis. The functional characteristics of these DEGs were identified through kyoto encyclopedia of genes and genomes (KEGG) pathway enrichment analysis. GO and KEGG pathway enrichment were conducted using Omicsmart, a real-time interactive online data analysis platform (http://www.omicsmart.com) 24,32.

### Cell proliferation assays

The effects of FOXM1 shRNA, FOXM1 cDNA, BUB1 shRNA, BUB1 cDNA, FDI-6, RCM-1, TST and BAY-1816032 (BAY) on the proliferation of HUH7 and HepG2 cells were evaluated using an MTT assay following the manufacturer's instructions. For rescue experiments, HUH7 cells were first infected with a FOXM1 shRNA lentiviral vector for 3 days, after which the virus-containing medium was replaced. These cells were then infected with a FOXM1 cDNA vector or BUB1 cDNA vector for an additional 3 days. After removing the virus-containing medium, cells were seeded at a density of 1×10^3^ cells/mL in a 96-well plate and cultured for 1, 2, 3, or 5 days before treatment. For FDI-6, RCM-1, TST, and BAY treatment experiments, HUH7 cells were seeded at 1×10^3^ cells/mL in 96-well plates, and were treated with different concentrations of these inhibitors for 1, 2, 3 or 5 days. Untreated cells served as the control. Following treatment, MTT solution was added to each well, and plates were incubated at 37 °C for 4.0 h. The medium was subsequently removed, and formazan crystals were dissolved in DMSO. Cell viability was determined by measuring absorbance at 570 nm using a Synergy NEO2 microplate reader (BioTek, USA). The relative MTT assay ratio was calculated as: relative ratio = A_Group, day_ / A_Control, 1_, where A_Group, day_ represents the absorbance of the experimental group at specific time points, and A_Control, 1_ denotes the absorbance of untreated cells after 1 day of culture 32.

### Drug combination assays

The combination ratios of BAY/FDI-6 were selected as 1:0.25, 1:0.5, 1:1 and 1:1.5. The combination ratios of BAY/TST were selected as 1:0.5 and 1:1.0. The combination ratios of BAY/TCM-1 were selected as 1:0.5 and 1:1.0. After cells were treated with FOXM1 inhibitor or/and BAY at different combination ratios for 3 days, the viabilities of HUH7 cells were detected by MTT assay 24. Untreated cells served as the control. The CI values were calculated by CompuSyn software using the equation CI=C_A,X_/IC_X,A_+C_B,X_/IC_X,B_. C_A,X_ and C_B,X_ represent the concentrations of BAY and FDI-6 that achieve to X% inhibition ratio in combination. IC_X, A_ and IC_X, B_ are the concentrations of BAY or FDI-6 that achieve to the same inhibition ratio alone. A combination index (CI) value less than 1.0 indicates a synergistic effect, a CI value equal to 1.0 indicates an additive effect, and a CI value greater than 1.0 indicates an antagonistic effect.

### Colony formation assay

All cells were seeded in 12-well plates at a density of 200 cells per well. For the silencing group, HUH7 and HepG2 cells stably expressing FOXM1 shRNA, FOXM1 cDNA, BUB1 shRNA, BUB1 cDNA, NC shRNA and NC cDNA were cultured in medium containing 0.5 μM puromycin for 14 days. For the drug treatment group, HUH7 and HepG2 cells were treated with various concentrations of FOXM1 inhibitor and/or BAY for 14 days. The culture medium was refreshed every 3 days to facilitate colony formation. After 14 days of treatment, colonies were fixed with cold methanol for 10 min, stained with 0.1% crystal violet for 10 min, and imaged using a Bio-Tek Live Cell Imaging System (Cytation 5, Vermont, USA). All experiments were independently repeated at least three times 24,32.

### Analysis of apoptosis and cell cycle

Cell apoptosis was analyzed using Annexin V-FITC and propidium iodide (PI) apoptosis detection kits (KeyGEN Biotech, Nanjing, China). Cell cycle was analyzed by a PI cell cycle detection kit (KeyGEN Biotech, Nanjing, Jiangsu, China). For the target silencing group, HUH7 cells were transfected with distinct lentiviral vectors for 3 days. For the drug treatment group, HuH-7 cells were treated with different concentrations of FDI-6, BAY, or their combination for 3 days. After treatment, cells were collected and stained with Annexin V-FITC and PI following the protocol of manufacturer to analyze cell apoptosis. Cellular DNA was stained with PI following the protocol of manufacturer. Cell apoptosis and cell cycle were detected by Agilent NovoCyte Quanteon (California, USA). Apoptosis data were analyzed by FlowJo 8.1 software, and the cell cycle data were analyzed by ModFit LT software 33.

### Alkaline comet assay

The effects of FOXM1, BUB1, and the FOXM1/BUB1 axis on DNA damage in HUH7 and HepG2 cells were performed as previously described using the alkaline comet assay, following the protocol of manufacturer (Comet Assay Kit, KeyGEN Biotech, Nanjing, China) 25. Following treatment, HCC cells (1×10⁴/mL) from each group were collected, combined with low melting point agarose at a 1:10 (v/v) ratio, layered onto slides, and lysed with lysis buffer at 4°C for 2.0 hours. Cells were then neutralized with an alkaline unwinding solution for another 30 min at room temperature. Following electrophoresis at 21 V for 30 min, the cells were stained with PI for 15 min at room temperature and observed with a Nikon-ECLIPSE 80i inverted fluorescence microscope (Tokyo, Japan). Five random images were captured per slide.

### Sphere formation assay

The effects of FOXM1, BUB1, and the FOXM1/BUB1 axis on cell stemness were performed using sphere formation assay as previously described. Following treatment, the cells were cultured in ultra-low attachment plates at a density of 1×10^4^ cells/mL. Cells in each group were maintained in serum-free DMEM/F12 medium supplemented with B27, 20 ng/mL epidermal growth factor (EGF), and 10 ng/mL basic fibroblast growth factor (bFGF) for 14 days. The culture medium was replenished every 4 days by replacing half of the volume to ensure optimal growth conditions. After 14 days of treatment, spheres were imaged using BioTek's live-cell imaging system (Cytation 5, Vermont, USA). Experiments were repeated at least three times 32.

### Migration and invasion assay

The effects of FOXM1, BUB1, and the FOXM1/BUB1 axis on cell migration and invasion of HUH7 and HepG2 cells were performed using Transwell plates as previously described 25,34. Following treatment, cells from each group were collected, counted, and assessed for migration and invasion capabilities. For cell migration, cells (2×10^4^/mL) were suspended in 200 μL of serum-free medium and seeded into the upper chamber, while the lower chamber contained medium supplemented with 10% FBS. For cell invasion, the upper chamber was coated with 20 μL of Matrigel. After Matrigel solidification, cells in 200 μL of serum-free medium were added to the upper chamber, with the lower chamber containing 10% FBS-supplemented medium. Following 48 h of incubation, cells were fixed with 4% formaldehyde for 30 min and stained with Giemsa for 15 min. Migrated or invaded cells on the outer surface of the upper chamber were visualized using a Nikon inverted fluorescence microscope (Ts2R-FL, Tokyo, Japan) according to the manufacturer's instructions. Five images per slide were randomly captured for analysis.

### Immunofluorescence (IF) stanning

After treatment, cells from each group were fixed with 4% formaldehyde, permeabilized using 0.2% (v/v) Triton X-100 in PBS, and blocked with 1% (w/v) BSA in PBS for 1.0 hour. They were then stained with anti-CD44 antibody or anti-γH2AX antibody overnight at 4 °C. Following anti-CD44 antibody staining, cells were incubated with Goat Anti-Rabbit IgG H&L conjugated to Alexa Fluor 647. After staining, cell nuclei were counterstained with 4,6-diamidino-2-phenylindole (DAPI). Fluorescence signals were visualized using a Carl Zeiss LSM900 laser confocal microscope (Jena, Germany), with five fields per sample analyzed for quantification 32,34.

### Q-PCR analysis

The effects of FOXM1, BUB1, and FOXM1/BUB1 axis on gene expression in HUH7 and HepG2 cells were analyzed by Q-PCR 24,32. After treatment, total RNA was extracted from cells using TRIzol reagent (Vazyme, Nanjing, China) according to the instructions of manufacturer. cDNA was synthesized with the HiScript II one-step RT-PCR kit (R223-01, Vazyme, Nanjing, China) using 1.0 μg of total RNA in a 20 μl reaction system. The resulting cDNA was diluted 1:2 in nuclease-free water, and 1.0 μl was used per Q-PCR reaction in triplicate. Q-PCR was carried out using ChamQ SYBR Q-PCR master mix (Q711-02, Vazyme, Nanjing, China) on a QuantStudio 3 real-time PCR detection system (Life Tech, New York, USA), including a non-template negative control. GAPDH was used to normalize mRNA expression levels. Primer sequences are listed in Table S1*.*

### Western blot analysis

After treatment, total proteins in HUH7 and HepG2 cells in each group were extracted using RIPA cell lysis buffer (Beyotime, Shanghai, China) supplemented with a protease/phosphatase inhibitor cocktail. Protein concentration was measured via the BCA assay, and equal amounts of protein were loaded for Western blot analysis. Briefly, equal quantities of total proteins were separated by SDS-PAGE and transferred onto a PVDF membrane (Millipore, Billerica, MA, USA). The membranes were blocked with 5% (w/v) skim milk, incubated with primary antibodies (listed in Supplementary Materials), and subsequently probed with secondary antibodies (1:2000 dilution) for detection. GAPDH was used as a loading control to normalize protein expression levels. Densitometric analysis was conducted using ImageJ software 24,32.

### Co-immunoprecipitation (Co-IP) assay

Total proteins were extracted from HUH7 cells using the lysis/washing buffer provided in the Protein A/G Magnetic IP/Co-IP kit (ACE Biotechnology, Nanjing, China). Protein A/G magnetic beads were incubated overnight at 4 °C with anti-FOXM1 antibody, anti-BUB1 antibody, or anti-IgG antibody to prepare the anti-FOXM1-Protein A/G magnetic beads, anti-BUB1-Protein A/G magnetic beads, and anti-IgG-Protein A/G magnetic beads, respectively. Proteins were quantified, adjusted to the same concentration, and then incubated with the prepared anti-FOXM1-Protein A/G magnetic beads, anti-BUB1-Protein A/G magnetic beads, or anti-IgG-Protein A/G magnetic beads at room temperature for 2.0 h. Unbound proteins were removed by washing with IP buffer after incubation. The resulting protein complexes were subjected to Western blot analysis to evaluate the interaction between FOXM1 and BUB1 32,33.

### ChIP-qPCR assay

The ChIP assay was performed using the Simple ChIP Plus Sonication Chromatin IP Kit (No. 56383, CST, USA) following the protocol of manufacturer. Briefly, HUH7 cells were crosslinked with 1% formaldehyde for 15 minutes. The reaction was quenched with glycine solution, followed by washing and lysing the cells in ChIP Sonication Cell Lysis Buffer. The nucleic fraction was isolated from the lysate and fragmented via sonication. Chromatin was immunoprecipitated by overnight incubation at 4 °C with either anti-FOXM1 antibody or anti-IgG antibody. Protein-DNA complexes were enriched and purified using ChIP-Grade Protein G beads. After purification, crosslinks were reversed by incubating the complexes with 5.0 M NaCl and Proteinase K for 2.0 h at 65 °C. DNA was purified using spin columns, resuspended in nuclease-free water, and analyzed by Q-PCR. The predicted FOXM1 binding sites in the BUB1 promoter region, as identified by the JASPAR online database, are located at -293 bp (sequence: GTAAACC) and -1429 bp (sequence:GTTAACA). The BUB1 primer sequences were as follows: forward primer (-293) #1 GACGCTGAATAGAAAACTGCC, reverse primer (-293) #1 GTTTCTTCTCCCCTTCGCTT; forward primer (-293) #2 GACTTGACCTCCGAGCAAC, reverse primer (-293) #2 CTGAACCGCAAACTAGAAGC; forward primer (-1429) #1 CGCTTTCTCATACAATGTCTGG, reverse primer (-1429) #1 AAGATAAATAGCTAGACGACCTT; forward primer (-1429) #2 TACATGCATCGGCTAACAGA, reverse primer (-1429) #2 AGATAAATAGCTAGACGACCT 32.

### Dual-luciferase reporter assay

The dual-luciferase reporter assay was employed to validate the binding site between FOXM1 and the BUB1 promoter. The -293 bp sequence in the BUB1 wild-type (BUB1-WT) promoter region is GTAAACC, and the -293 bp sequence in the BUB1 mutant (BUB1-Mut) promoter region is CATTTGG. Both BUB1-WT and BUB1-Mut promoter sequences were cloned into the pGL3-basic plasmid, which contains the firefly luciferase reporter gene. FOXM1 cDNA was cloned into the the pcDNA3.1 plasmid. The pRL-TK plasmid, containing the Renilla luciferase gene, was used as an internal control. All three plasmids were constructed and purified by Beijing Qingke Biotechnology Co., Ltd. (Beijing, China). For the assay, HUH7 cells were seeded into 12-well plates and co-transfected with 1.5 μg of the pGL3-recombinant plasmid (either BUB1-WT or BUB1-Mut), 1.5 μg of the FOXM1 overexpression (OE) plasmid, and 0.3 μg of pRL-TK plasmid per well. At 6 hours post-transfection, the transfection medium was replaced with complete culture medium with 8% fetal bovine serum (FBS), and cells were cultured for an additional 48 h. Subsequently, cells were lysed, and 200 μL of lysate per well was collected for luciferase activity measurement. Firefly and Renilla luciferase activities were quantified using the Firefly & Renilla Luciferase Reporter Gene Assay Kit (Guangzhou Biolight Biotechnology, Guangzhou, China), according to the manufacturer's instructions.

### Animal care and treatment

All animal experiments were conducted in compliance with institutional guidelines for the care and use of laboratory animals and were approved by the Animal Experiment Ethics Committee of Lanzhou University (Approval No. LDYYLL2024-92). Six-week-old athymic BALB/c-nude mice (18-19 g) were supplied by the Model Animal Institute of Nanjing University (Nanjing, Jiangsu, China). Mice were housed under controlled conditions at 25 °C with 60-70% relative humidity and a 12 h light/dark cycle. HUH7 cells stably expression NC shRNA, FOXM1 shRNA, FOXM1 cDNA, and BUB1 shRNA (1×10⁷ cells/mL) were injected into the right flank of each mouse. For target silencing, tumor volume and body weight were monitored after the average xenograft tumor volume in the NC KD group reached 50 mm³. Following 35 days of observation, mice were euthanized to assess tumor weights in each group. For drug treatment, the tumor-bearing mice were randomly divided into four groups (n = 5/group) after the average tumor volume in NC KD group reached to 50 mm^3^. 20 mg/kg FDI-6 was administered by intraperitoneal injection for 21 consecutive days. Tumor volume was recorded every two days. After 21 days of treatment, the mice were killed to detect the weight of tumors in each group. For drug combination assay, the tumor-bearing mice were randomly divided into two groups (n = 5/group) after the average tumor volume in model group reached to 50 mm^3^. For single-dose administration, 20 mg/kg of FDI-6 or 20 mg/kg BAY was administered intraperitoneally for 21 days. For sequential administration, 20 mg/kg FDI-6 was intraperitoneally injected for 10 days, and then starting from the 12th day, 20 mg/kg BAY was intraperitoneally injected for another 10 days. The tumor volume was recorded every two days. Tumor volume was measured with a vernier caliper and calculated using the formula (ab^2^)/2, where a and b represent the length and width of the tumor, respectively 24,33.

### Acute toxicity assay

Seven-week-old Swiss ICR mice (female/male, weighing 18-20 g) were supplied by the Laboratory animal center of Lanzhou University (Lanzhou, Gansu, China). Swiss ICR mice were divided into three groups (n = 10/group, female = 5, male = 5). Menstruum, FDI-6 (100 mg/kg), BAY (100 mg/kg), and their combination were administered via intraperitoneal injection on the first day. Following treatment, the body weight and general health status of the ICR mice were monitored and recorded daily for seven consecutive days 34.

### Hematoxylin and eosin (H&E) staining

The lung, liver, heart, kidney, and spleen tissue samples from HUH7 tumor xenograft model mice were fixed in 4% paraformaldehyde, dehydrated in ethanol, cleared in xylene, embedded in paraffin, and sectioned longitudinally at 4.0 μm thickness. Paraffin-embedded sections were stained with H&E following the manufacturer's protocol (Beyotime, Shanghai, China). Tissue samples from each group were examined using a DM6B-positive fluorescence microscope (Leica, Frankfurt, Germany), with five randomly selected images captured per slide 24,32.

### Immunohistochemistry (IHC) staining

Tumor tissues and adjacent normal tissues were collected from 11 HCC patients at The Frist Hospital & Clinical Medical School of Lanzhou University, with approval from the Medical Ethics Committee of the Frist Hospital of Lanzhou University (LDYYLL2024-153). All patients provided informed consent prior to sample collection. Both xenograft tumors and HCC patient-derived tissues were embedded in paraffin and then cut into longitudinal sections. The paraffin-embedded sections were incubated with 0.3% hydrogen peroxide for 30 min to block endogenous peroxidase activity, followed by incubation with 1.0% BSA for blocking. After blocking, the sections were incubated with anti-FOXM1 antibody, anti-BUB1 antibody, or anti-Ki67 antibody overnight at 4 °C, then with a secondary antibody for 1.0 h at room temperature, and counterstained with hematoxylin for 1.0 min. Each group was analyzed using a DM6B fluorescence microscope (Leica, Frankfurt, Germany). Five images were randomly captured per slide. The percentage of stained dots was quantified using ImageJ software 24,32.

### Statistical analysis

The data were analyzed using SPSS 19.0 and are presented as means ± SD from at least three independent experiments. Differences between treatment groups were assessed by one-way ANOVA and Student's t-test. A P value of < 0.05 was considered statistically significant.

## Results

### FOXM1 promotes the malignant proliferation of HCC cells

**Figure 1:**
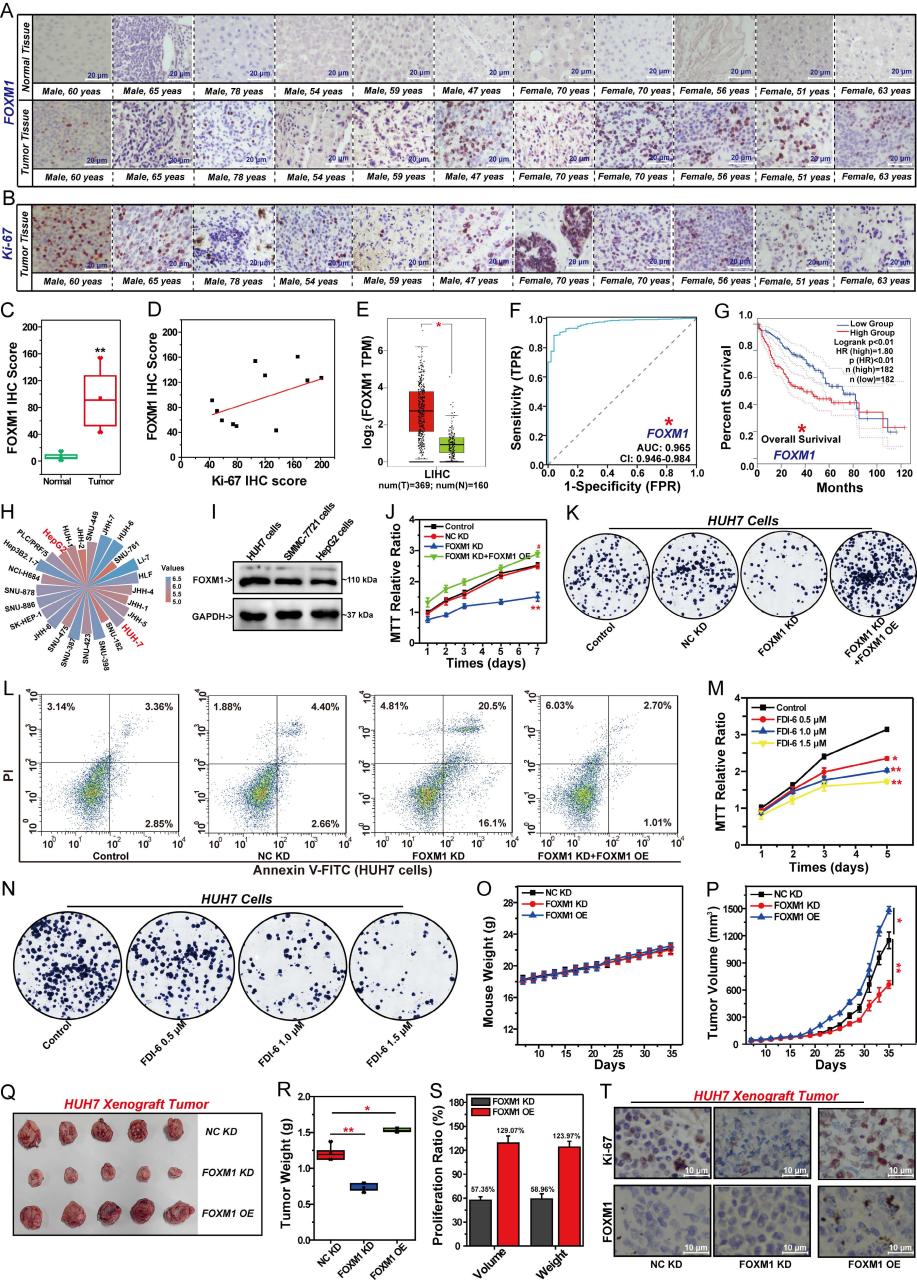
** FOXM1 promotes the proliferation of HCC cells *in vitro*. (A)** The expression of FOXM1 in tumor tissues and adjacent tissues of patients with HCC analyzed by IHC. **(B)** The expression of Ki-67 in tumor tissues of patients with HCC analyzed by IHC. **(C)** The differential expression of FOXM1 between tumor tissues and adjacent tissues of patients with HCC analyzed by IHC. **(D)** Correlation analysis of FOXM1 and Ki-67 IHC scores in tumor tissues of patients with HCC. **(E)** Box plots for the differential expression of FOXM1 between LIHC tissues and adjacent tissues in TCGA. **(F)** ROC curves for the relationship between FOXM1 expression and the prognosis of HCC patients. **(G)** Correlation between FOXM1 expression levels and survival of HCC patients. **(H)** Distribution of FOXM1 mRNA expression in HCC cell lines. **(I)** Expression of FOXM1 in HUH7, HepG2, and SMMC-7721 cells analyzed by Western blot. **(J)** Effect of FOXM1 shRNA and FOXM1 cDNA on the viability of HUH7 cells. **(K)** Effects of FOXM1 on the formation of HUH7 colonies. **(L)** Effects of FOXM1 on the apoptosis of HUH7 cells. **(M)** Effects of FDI-6 on the proliferation of HUH7 cells. **(N)** Effects of FDI-6 on the formation of HUH7 colonies. **(O)** Effects of FOXM1 shRNA and FOXM1 cDNA on mouse weight. **(P)** Effects of FOXM1 shRNA and FOXM1 cDNA on tumor volume. **(Q)** The photos of tumor nodules in each group. **(R)** Effects of FOXM1 shRNA and FOXM1 cDNA on tumor weight. **(S)** Effects of FOXM1 shRNA and FOXM1 cDNA on tumor proliferation. **(T)** The expression of Ki-67 and FOXM1 in xenograft tumor analyzed by IHC. Images were randomly selected from five replicates. Data from three independent experiments were analyzed by one-way ANOVA: ^*^P<0.05, ^**^P<0.01 vs. control group; ^#^P<0.05,^ ##^P<0.01 vs. FOXM1 KD group.

The oncogenic transcription factor FOXM1 has been confirmed to be closely associated with the progression of various cancers 9-12. To evaluate whether FOXM1 is a potential therapeutic target in HCC, we initially examined the differential expression of FOXM1 in tumor tissues compared to adjacent normal tissues from 11 HCC patients (Fig. 1A). Additionally, we analyzed the expression levels of Ki-67 within the corresponding tumor tissues (Fig. 1B). Statistical analysis revealed that FOXM1 expression was significantly higher in the tumor tissues of these 11 patients than in the adjacent normal tissues (Fig. 1C, Table S2). Subsequently, we assessed the correlation between FOXM1 and Ki-67 expression levels in the tumor tissues of these patients. The results demonstrated a positive correlation between FOXM1 and Ki-67 IHC scores, suggesting that high FOXM1 expression is associated with increased tumor malignancy and poor prognosis in HCC patients (Fig. 1D, Table S2). Indeed, we found that the expression of FOXM1 was significantly higher in HCC tissues compared to adjacent normal tissues, as shown in the Human Protein Atlas database (Fig. S1A, B). To validate these findings, we analyzed the differential expression of FOXM1 in LIHC tissues and adjacent normal tissues from TCGA database, which also demonstrated significantly higher FOXM1 expression in tumor tissues (Fig. 1E). Moreover, FOXM1 expression exhibited an increasing trend across clinical stages I to III and was overexpressed in all stages examined (Fig. S1C). Further analysis of the effect of FOXM1 expression on HCC prognosis was conducted using ROC curves. The AUC value of the ROC curve reflects the correlation between FOXM1 expression and cancer prognosis 35. The AUC value for FOXM1 was 0.96, confirming that FOXM1 expression was significantly associated with poor prognosis in HCC (Fig. 1F). Subsequent analysis of the relationship between FOXM1 expression and HCC patient survival revealed that increased FOXM1 expression significantly reduced both OS and DFS (Fig. 1G, S1D). These findings suggest that FOXM1 overexpression is closely linked to malignant progression and poor prognosis in HCC. Based on these observations, we analyzed the mRNA expression distribution of FOXM1 in HCC cell lines, which showed high expression levels in HUH7 and HepG2 cells (Fig. 1H, S1E). Western blot and Q-PCR results further confirmed the increased expression level of FOXM1 in HUH7 and HepG2 cells (Fig. 1I, S1F, G). We therefore selected these two cell lines for subsequent studies. The effects of FOXM1 on the proliferation in HUH7 cells were evaluated by MTT assay at different time points (Fig. 1J). FOXM1 shRNA significantly inhibited the proliferation of HUH7 cells. The proliferation ability of the FOXM1 knockdown (KD) group was significantly lower than that of the control and NC KD groups. The rescue experiment demonstrated that overexpression of FOXM1 could restore the proliferation ability of FOXM1 KD cells, which confirmed that FOXM1 is a key regulatory factor for the proliferation of HCC cells. Colony formation assays further validated that FOXM1 shRNA suppressed the formation of HUH7 colonies, while FOXM1 cDNA reversed this inhibitory effect (Fig. 1K, Fig. S1H). Similarly, apoptosis assays revealed that FOXM1 shRNA promoted the apoptosis of HUH7 cells. FOXM1 silencing increased the apoptosis rate from 7.1% to 36.6% compared to the NC KD group, which was reduced to 3.7% after FOXM1 overexpression in the rescue group (Fig. 1L, S1I). Subsequently, we analyzed the effect of the FOXM1 inhibitor FDI-6 on the malignant proliferation of HUH7 cells. The results showed that 1.0 μM of FDI-6 significantly inhibited the proliferation and colony formation of HUH7 cells, and promoted the apoptosis of HUH7 cells (Fig. 1M, N, S1J-L).

Based on the above research results, we established stable FOXM1-knockdown HUH7 cells and stable FOXM1-overexpression HUH7 cells to evaluate the effect of FOXM1 on HCC cell growth* in vivo* (Fig. 1O-S, S1M). FOXM1-knockdown HUH7 cells, FOXM1-overexprssion HUH7 cells and NC-knockdown HUH7 cells were used to generate cell line-derived xenograft (CDX) tumor. After 35 days of observation, FOXM1 knockdown or overexpression showed no impact on mouse body weight (Fig. 1O). FOXM1 shRNA significantly suppressed both the volume and weight of HUH7 xenograft tumors. Compared with the NC KD group, the tumor volume and weight inhibition rates in the FOXM1 KD group were 57.4% and 59.0%, respectively (Fig. 1P-S). Overexpression of FOXM1 significantly promoted the proliferation of HUH7 xenograft tumors (Fig. 1P-S). IHC analysis revealed that the Ki-67 level in the FOXM1 KD group was significantly decreased, while the Ki-67 level in the FOXM1 OE group was significantly increased. This confirmed the promoting effect of FOXM1 on the malignant proliferation of HCC (Fig. 1T, S1M).

### FOXM1 regulates genes-related to DNA repair, cell cycle, stemness and EMT

**Figure 2:**
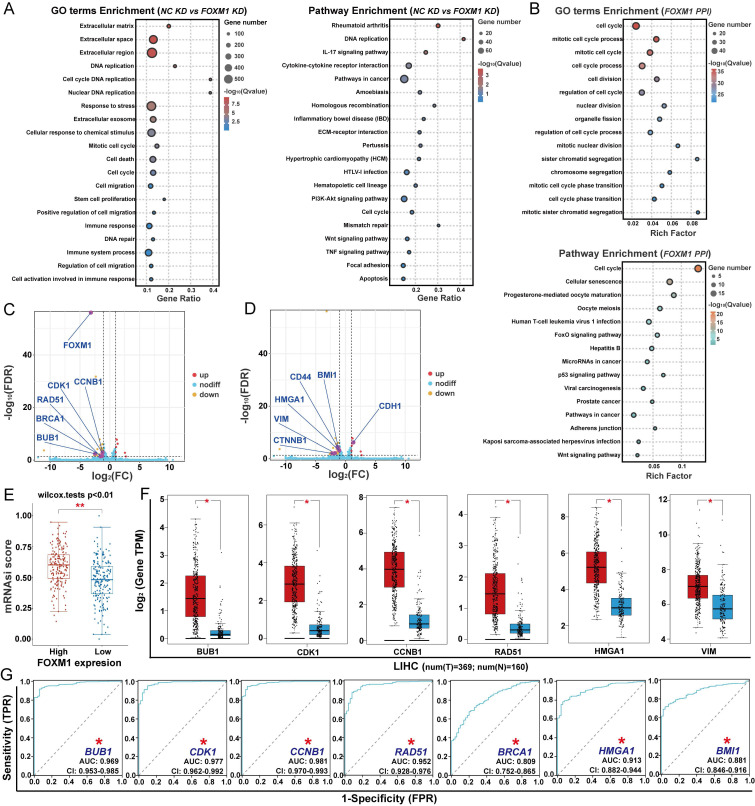
** FOXM1-regulated genes involve in DNA repair, cell cycle, stemness and EMT. (A)** GO function and KEGG pathway enrichment analysis of DEGs between FOXM1 KD and NC KD in HUH7 cells. **(B)**. GO function and KEGG pathway enrichment analysis of top 50 targets in the PPI network of FOXM1. **(C)**. Volcano plot for the DEGs involved in DNA repair and cell cycle analyzed by RNA-seq. **(D)** Volcano plot for the DEGs involved in cell stemness, invasion and migration. **(E)**. The relationship between FOXM1 expression and HCC cell stemness in TCGA. **(F)**. Differential expression of genes between LIHC tissues and adjacent tissues in TCGA. **(G)**. ROC curves for the relationship between gene expression and HCC prognosis. Data were statistically analyzed using one-way ANOVA: ^*^P<0.05 compared with adjacent tissues.

To clarify the mechanism of FOXM1 in regulating the proliferation of HCC cells, we used RNA-seq to analyze its effects on gene expression in HUH7 cells (Fig. 2A). Knockdown of FOXM1 induced upregulation of 89 DEGs and downregulation of 547 DEGs in HUH7 cells. GO functional enrichment and KEGG pathway analyses revealed that FOXM1-regulated DEGs were primarily associated with DNA replication, DNA repair, cell death, cell cycle, cell migration and cell stemness (Fig. 2A). Further GO functional and KEGG pathway analyses of the FOXM1 protein-protein interaction (PPI) network indicated that FOXM1 indeed participates in the regulation of cell cycle, metastasis and stemness-related signaling pathways (Fig. 2B, Table S3). Comparative analysis between the NC KD group and FOXM1 KD group identified 11 DEGs based on GO function and KEGG pathway enrichment (Fig. 2C, D). Among them, radiation defense 51 (RAD51) and breast cancer susceptibility gene 1 (BRCA1) are involved in DNA repair, while cyclin-dependent kinase 1 (CDK1), cyclin B1 (CCNB1), and BUB1 are involved in the cell cycle process (Fig. 2C). B lymphoma Mo-MLV insertion region 1 homolog (BMI1), high mobility group A1 (HMGA1), cluster of differentiation 44 (CD44), and β-catenin (CTNNB1) are involved in regulating cell stemness, while vimentin (VIM), and E-cadherin (CDH1) are involved in epithelial-mesenchymal transition (EMT)-mediated cell invasion and migration (Fig. 2D). Among these DEGs, CDK1, CCNB1, BUB1 and CTNNB1 are the key factors in the FOXM1 PPI network. Subsequently, we used the OCLR algorithm to analyze the relationship between FOXM1 expression levels and the stemness of LIHC in the TCGA database. We found that the higher the expression of FOXM1, the higher the degree of HCC cell stemness (Fig. 2E). Differential expression analysis of TCGA database data showed that BUB1, CDK1, CCNB1, RAD51, HMGA1 and VIM were overexpressed in LIHC (Fig. 2F, S2A). The subsequent prognostic analysis indicated that the expressions of the three genes related to the cell cycle, two genes related to DNA repair, HMGA1 and BMI1 were positively correlated with the poor prognosis of patients with HCC (Fig. 2G, S2B). In summary, FOXM1 promotes HCC malignancy by regulating genes involved in DNA damage repair, cell cycle progression, stemness, and EMT.

### Inhibition of FOXM1 suppresses DNA repair, and cell cycle progression in HCC cells

**Figure 3:**
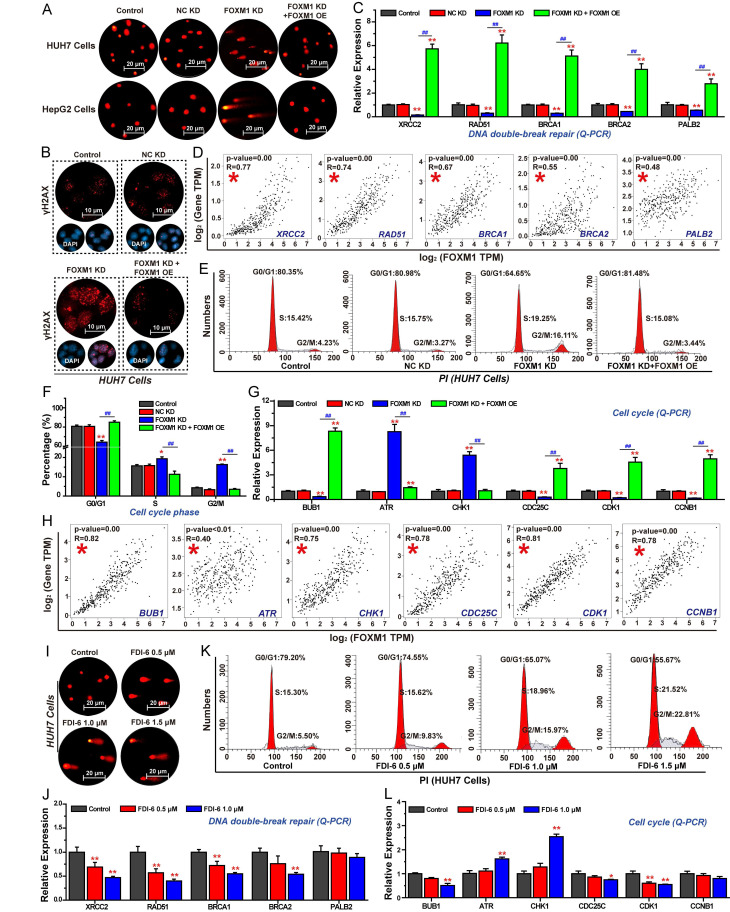
** FOXM1 promotes DNA repair and G2/M progression on HCC cells. (A)** Effects of FOXM1 on DNA damage in HUH7 and HepG2 cells analyzed by comet assay. **(B)** Effects of FOXM1 on γH2AX expression analyzed by IF. **(C)** Q-PCR analysis of the effects of FOXM1 on the expression of DNA repair-related genes. **(D)** Analysis of the correlation between FOXM1 and DNA repair-related gene expressions in LIHC. **(E)** Effects of FOXM1 on cell cycle progression in HUH7 cells. **(F)** percentage of cell cycle at G0/G1, S, and G2/M phases. **(G)** Q-PCR analysis of the effects of FOXM1 on the expression of genes regulating G2/M transition. **(H)** Analysis of the correlation between FOXM1 and cell cycle related gene expressions in HCC. **(I)** Effects of FDI-6 on DNA damage in HUH7 cells. **(J)** Effects of FDI-6 on the expression of DNA-repair related genes in HUH7 cells. **(K)** Effects of FDI-6 on cell cycle progression in HUH7 cells. **(L)** Effects of FDI-6 on cell cycle-related genes in HUH7 cells. Images were randomly selected from five replicates. Data from three independent experiments were statistically analyzed using one-way ANOVA: ^*^P < 0.05, ^**^P < 0.01 vs. NC KD group; ^#^P < 0.05, ^##^P < 0.01 vs. FOXM1 KD group.

Based on RNA-seq analysis, we firstly investigated the effects of FOXM1 on DNA damage repair, and cell cycle progression in HCC cells. The comet assay, commonly used to assess intracellular DNA damage, revealed that longer tail lengths and greater numbers of tailed cells indicated more severe DNA damage 36. Results demonstrated that inhibition of FOXM1 expression significantly promoted DNA damage in HUH7 and HepG2 cells (Fig. 3A). As γ-H2AX recognizes DNA double-strand breaks, its levels increase with DNA damage severity 37. The results of IF confirmed that knockdown of FOXM1 significantly enhanced γ-H2AX expression in HUH7 cells (Fig. 3B). Further Q-PCR results showed that silencing FOXM1 expression in HUH7 cells significantly inhibited the expression of X-ray repair cross-complementing protein 2 (XRCC2), RAD51, BRCA1, and partner and localizer of BRCA2 (PALB2), thereby impairing DNA double-strand breaks repair (Fig. 3C). Rescue experiments demonstrated that overexpression of FOXM1 reversed the expression of these genes. Subsequent correlation analysis showed that the expressions of these five DNA double-strand break repair-related genes were positively correlated with the expression of FOXM1 in LIHC (Fig. 3D). We also analyzed the effects of FOXM1 on HCC cell cycle progression. Compared with the control group and the NC KD group, knockdown of FOXM1 arrested the cell cycle of HUH7 cells in the G2/M phase, and overexpression of FOXM1 reversed this arresting effect (Fig. 3E, F). Q-PCR results further confirmed that the knockdown of FOXM1 promoted the expression of ataxia telangiectasia and Rad3-related (ATR), and checkpoint kinase 1 (CHK1), inhibited the expression of cell division cycle 25C (CDC25C), CDK1, CCNB1 and BUB1, thereby inhibiting the transition process from G2 phase to M phase (Fig. 3G). Further correlation analysis showed that the expressions of these six-cell cycle-related genes were positively correlated with the expression of FOXM1 in LIHC (Fig. 3H). Subsequently, we analyzed the effect of the FOXM1 inhibitor FDI-6 on DNA damage repair and cell cycle progression in HUH7 cells. The results showed that 1.0 μM of FDI-6 significantly induced DNA damage and arrested cell cycle at G2/M phase (Fig. 3I, J, S3). Q-PCR results confirmed that by inhibiting the transcriptional function of FOXM1 using FDI-6, the expression of genes related to DNA repair and cell cycle was also regulated, thereby inhibiting the repair of DNA double-strand breaks and blocking the transition from the G2 phase to the M phase (Fig. 3K, L).

### Inhibition of FOXM1 impairs cell stemness, invasion, and migration in HCC cells

**Figure 4:**
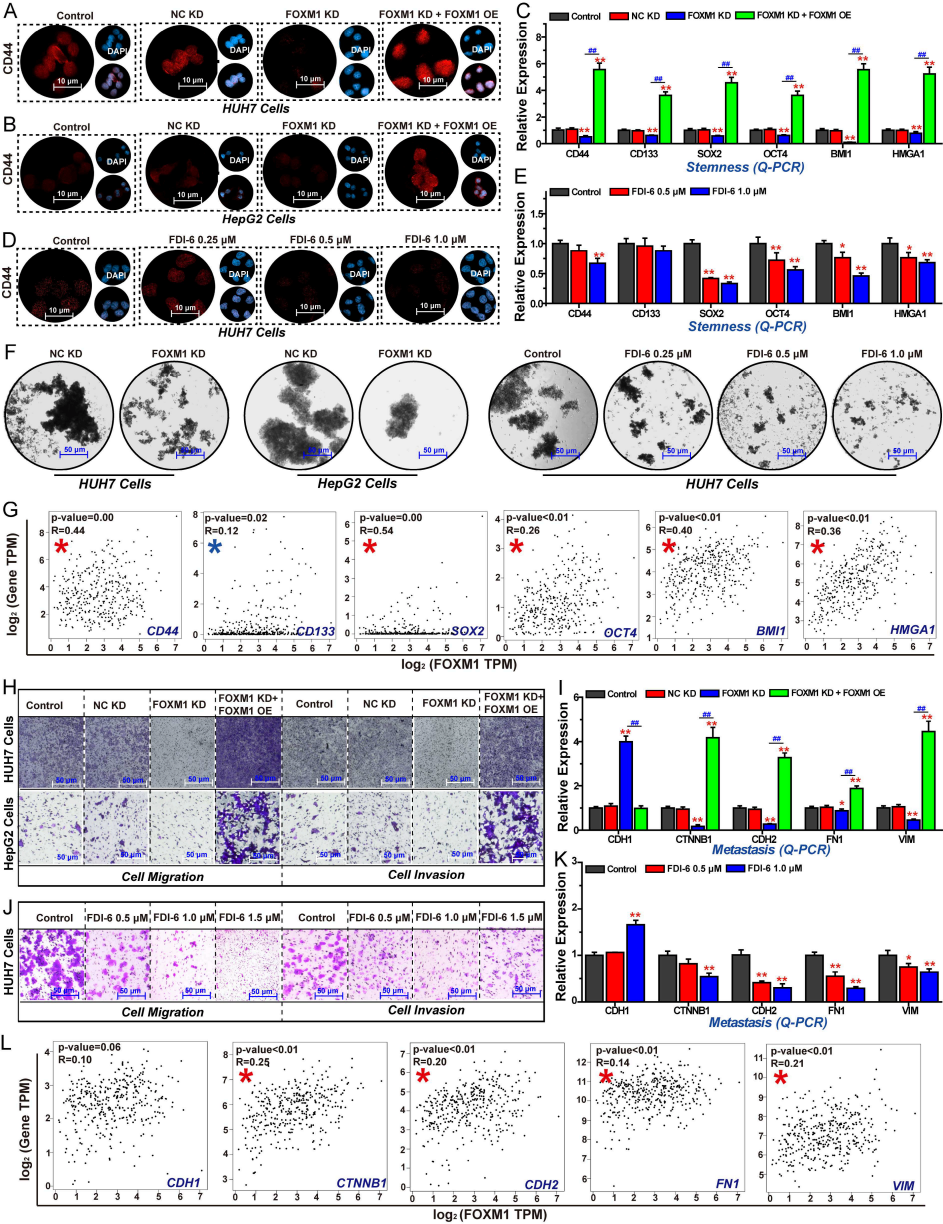
** FOXM1 drives cell stemness, invasion, and migration in HCC cells. (A)** Effects of FOXM1 on CD44 expression analyzed by IF in HUH7 cells. **(B)** Effects of FOXM1 on CD44 expression in HepG2 cells. **(C)** Effects of FOXM1 on the expression of stemness-related genes in HUH7 cells. **(D)** Effects of FDI-6 on CD44 expression in HUH7 cells. **(E)** Effects of FDI-6 on the expression of stemness-related genes in HUH7 cells. **(F)** Effects of FOXM1 shRNA and FDI-6 on the formation of HUH7 three-dimensional spheres. **(G)** Analysis of the correlation between FOXM1 and cell cycle-related gene expressions in HCC. **(H)** Effects of FOXM1 on the invasion and migration of HUH7 and HepG2 cells. **(I)** Q-PCR analysis of the effects of FOXM1 on EMT-related gene expression in HUH7 cells. **(J)** Effects of FDI-6 on the invasion and migration of HUH7 cells. **(K)** Q-PCR analysis of the effects of FDI-6 on EMT-related gene expression in HUH7 cells. **(L)** Analysis of the correlation between FOXM1 and EMT-related gene expressions in HCC. Images were randomly selected from five replicates. Data from three independent experiments were statistically analyzed using one-way ANOVA: ^*^P < 0.05, ^**^P < 0.01 vs. NC KD group; ^#^P < 0.05, ^##^P < 0.01 vs. FOXM1 KD group.

Additionally, we investigated the effects of FOXM1 on cell stemness, migration, and invasion in HCC cells. The results of IF assays revealed that knockdown of FOXM1 in both HUH7 and HepG2 cells, the expression of the stem cell biomarker CD44 was significantly reduced. Restoring the expression of FOXM1 could reverse this inhibitory effect, indicating that FOXM1 promotes HCC cell stemness (Fig. 4A, B). Similarly, the FOXM1 inhibitor FDI-6 significantly suppressed CD44 expression (Fig. 4C). The subsequent Q-PCR experiment results showed that silencing FOXM1 expression in HUH7 cells significantly inhibited the expression of CD44, cluster of differentiation 133 (CD133), SRY-box transcription factor 2 (SOX2), octamer-binding transcription factor 4 (OCT4), BMI1, and HMGA1, thereby impairing the stemness of HCC cells (Fig. 4D). FDI-6 also significantly inhibited the expression of these genes, except for CD133 expression (Fig. 4E). Subsequently, we employed the sphere formation assay to validate the role of FOXM1 in regulating HCC cell stemness. The sphere formation assay is used to evaluate cancer cell stemness, where a higher number of three-dimensional spheres indicates greater proliferative capacity and stemness levels in the corresponding cells 38. Compared with NC KD group, the numbers of HUH7 and HepG2 spheres were significantly reduced in the FOXM1 KD group. FDI-6 also significantly inhibited the formation of HUH7 spheres (Fig. 4F, S4A, B). Correlation analysis further showed that the expressions of these six stemness-related genes were positively correlated with the expression of FOXM1 in LIHC (Fig. 4G). Finally, we analyzed the impact of FOXM1 on HCC cell invasion and migration. The results showed that silencing FOXM1 expression in HUH7 and HepG2 cells significantly inhibited cell invasion and migration. Restoring FOXM1 expression could reverse this inhibitory effect, indicating that FOXM1 promotes HCC cell migration and invasion (Fig. 4H, S4C). Knockdown of FOXM1 suppressed the expression of N-cadherin (CDH2), CTNNB1, fibronectin 1 (FN1), and VIM while promoting CDH1 expression, thereby inhibiting EMT-mediated cell migration and invasion. Restoring FOXM1 expression could reverse the expression of EMT-related genes (Fig. 4I). 1.0 μM FDI-6 also significantly inhibited the migration and invasion of HUH7 cells (Fig. 4J, S4D). Q-PCR results revealed that FDI-6 regulated EMT-related genes to inhibit HCC cell invasion and migration (Fig. 4K). In summary, FOXM1 is a critical target promoting HCC cell proliferation, stemness, invasion, and migration.

### FOXM1 regulates BUB1 expression to promote HCC cell proliferation

**Figure 5:**
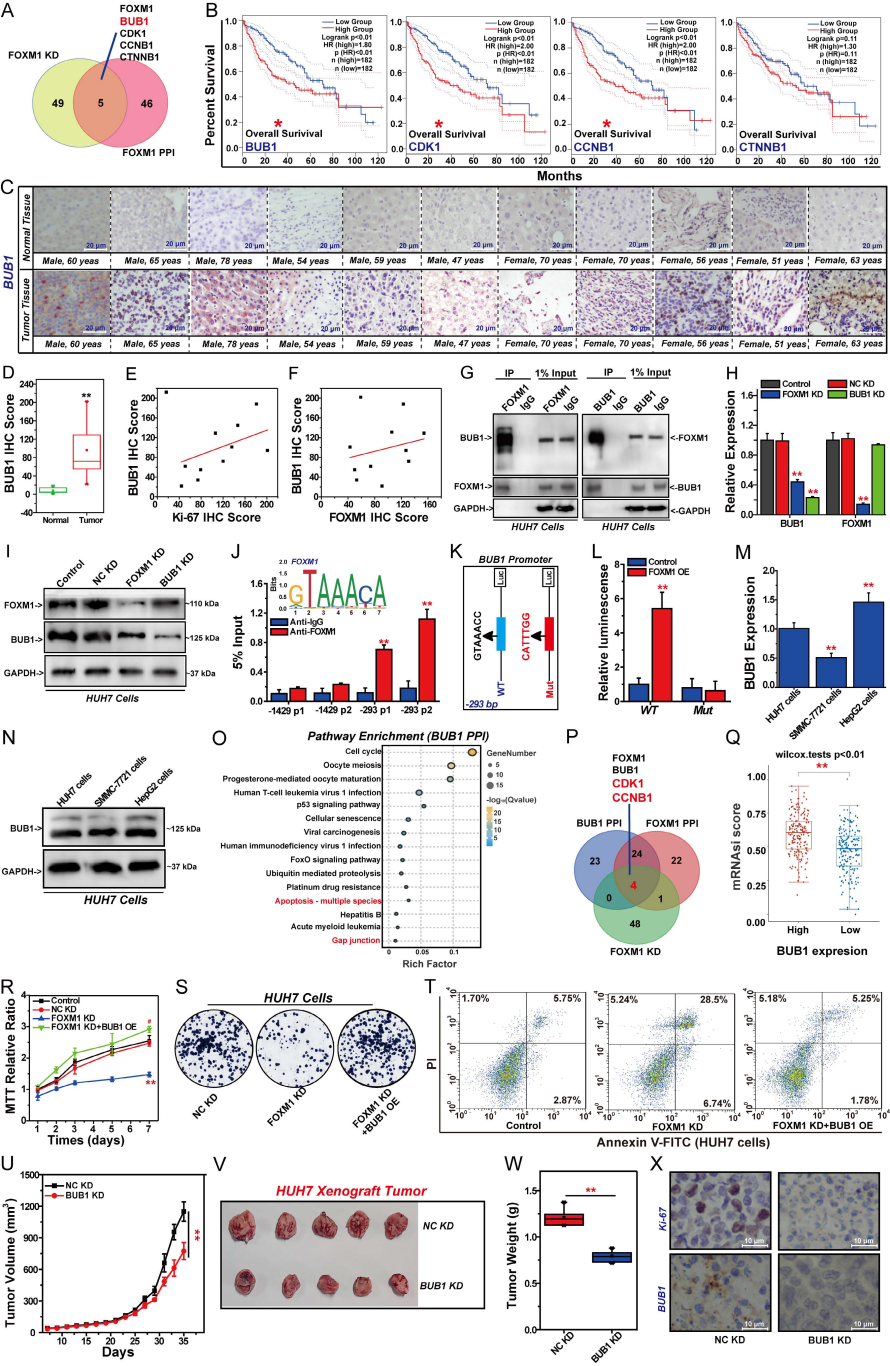
** FOXM1 promotes BUB1 expression at transcriptional level. (A)** Venn diagram of overlapping genes identified by RNA-seq and FOXM1 PPI network. **(B)** Analysis the association between key gene expression and HCC patient survival. **(C)** The expression of BUB1 between HCC tissues and adjacent tissues from clinical patients analyzed by IHC. **(D)** IHC scores of BUB1 between HCC tissues and adjacent tissues from clinical patients. **(E)** Correlation analysis of BUB1 and Ki-67 IHC scores in tumor tissues of patients with HCC. **(F)** Correlation analysis of BUB1 and FOXM1 IHC scores in tumor tissues of patients with HCC. **(G)** Co-IP analysis of the interaction between FOXM1 and BUB1 in HUH7 cells. **(H)** Effects of FOXM1 shRNA and BUB1 shRNA on the expression of FOXM1 and BUB1 analyzed by Q-PCR. **(I)** The expression of FOXM1 and BUB1 analyzed by Western blot. **(J)** ChIP-qPCR analysis of the binding of FOXM1 and BUB1 promoter in HUH7 cells. **(K)** The sequences at the -293 bp of the BUB1-WT promoter and the BUB1-Mut promoter. **(L)** The binding of FOXM1 and BUB1 promoter at the -293 bp sequence GTAAACC analyzed by dual luciferase reporter assay. **(M)** Q-PCR analysis of BUB1 expression in different HCC cells. **(N)** Western blot analysis of BUB1 expression in different HCC cells. **(O)** KEGG pathway enrichment analysis of top 50 targets in the PPI network of BUB1. **(P)** Venn diagram of overlapping genes identified by RNA-seq, FOXM1 PPI network, and BUB1 PPI network. **(Q)** correlation analysis of BUB1 expression and LIHC cell stemness in TCGA. **(R)** Effect of BUB1 on the inhibitory role of FOXM1 shRNA in the proliferation of HUH7 cells. **(S)** Effects of BUB1 on the inhibition of FOXM1 shRNA in HUH7 colony formation. **(T)** Effects of BUB1 on the promotion of FOXM1 shRNA in HUH7 cell apoptosis. **(U)** Effects of BUB1 shRNA on HUH7 xenograft tumor volumes. **(V)** Photos of HUH7 xenograft tumors in each group. **(W)** Effects of BUB1 shRNA on HUH7 xenograft tumor weight. **(X)** Effects of BUB1 shRNA on Ki-67 and BUB1 expression in each group. Images were randomly selected from five replicates. Data from three independent experiments were analyzed by one-way ANOVA: ^*^P<0.05, ^**^P<0.01 vs. NC KD group, ^#^P < 0.05, ^##^P < 0.01 vs. FOXM1 KD group.

Silencing the expression of FOXM1 in HUH7 cells suppressed the expression of BUB1, CDK1, CCNB1, and CTNNB1. These four genes are key factors in the PPI network of FOXM1, indicating that these four targets play an important role in the oncogenic function of FOXM1 (Fig. 5A). Indeed, the overexpression of BUB1, CDK1 and CCNB1 leads to a significant reduction in the OS period of patients with HCC, driving the malignant progression of HCC (Fig. 5B). Indeed, existing studies have investigated the regulatory interactions among FOXM1, BUB1, and CDK1, confirming that the FOXM1/CDK1/CCNB1 pathway plays a critical role in cell cycle regulation and drives malignant progression of tumors 39-41. However, the regulatory relationship between FOXM1 and BUB1 remains unclear. IHC analysis of tumor tissues and adjacent normal tissues from 11 HCC patients demonstrated that BUB1 expression was significantly upregulated in tumor tissues (Fig. 5C, D, Table S2). IHC results further confirmed that the expression level of BUB1 in tumor tissues was positively correlated with that of Ki-67, indicating that BUB1 is closely associated with poor prognosis in patients with HCC (Fig. 5E, Table S2). Moreover, IHC results demonstrated a positive correlation between BUB1 and FOXM1 expression in tumor tissues, suggesting that these two proteins may cooperatively contribute to HCC progression (Fig. 5F, Table S2). Indeed, Co-IP assays showed a direct interaction between FOXM1 and BUB1 in HUH7 cells (Fig. 5G). Therefore, we investigated the regulatory relationship between FOXM1 and BUB1. Q-PCR and Western blot results demonstrated that knockdown of FOXM1 significantly reduced BUB1 expression at both transcriptional and protein levels. Conversely, knockdown of BUB1 did not significantly regulate FOXM1 expression (Fig. 5H, I, S5A). These findings suggest that BUB1 is a downstream target of FOXM1. As a critical transcription factor, FOXM1 regulates downstream targets at the transcriptional level 9. To determine whether BUB1 is directly regulated by FOXM1 at transcriptional level, we analyzed FOXM1 ChIP-seq data from the cistrome data browser. Distinct peaks at the BUB1 promoter were observed, suggesting a direct transcriptional regulation (Fig. S5B). ChIP-qPCR results demonstrated that FOXM1 directly binds to the BUB1 promoter at -293 bp (sequence: GTAAACC), indicating that BUB1 is a direct transcriptional target of FOXM1 (Fig. 5J). Subsequent dual-luciferase reporter assays showed that FOXM1 activates BUB1 expression at the transcriptional level. Notably, when the -293 bp sequence in the BUB1 promoter was mutated from GTAAACC to CATTTGG, FOXM1-mediated transcriptional activation of BUB1 was abolished (Fig. 5K, L).

These findings confirm that FOXM1 promotes BUB1 expression by binding to the GTAAACC motif in the BUB1 promoter region. Subsequent studies revealed that BUB1 is also highly expressed in HUH7 and HepG2 cells (Fig. 5M, N, S5B). The GO function and KEGG pathway enrichment analyses of the top 50 targets in the BUB1 PPI network indicated that BUB1 is involved in regulating the cell cycle, apoptosis, cell migration, and drug resistance (Fig. 5O, S5C, Table S4). CDK1 and CCNB1 are common genes in FOXM1 PPI network and BUB1 PPI network (Fig. 5P). Cell stemness is closely related to the drug resistance and cancer recurrence 42. Therefore, we analyzed the relationship between BUB1 expression and the stemness of HCC cells. The results showed that the overexpression of BUB1 could promote HCC cell stemness (Fig. 5Q). Therefore, we speculate that FOXM1 exerts a promoting effect on the malignant proliferation of HCC cells by regulating BUB1 expression. Rescue experiments were conducted to analyze the role of BUB1 in regulating FOXM1 functionality. MTT results showed that overexpression of BUB1 could reverse the inhibitory effect of FOXM1 shRNA on the proliferation of HUH7 cells (Fig. 5R). Colony formation assays confirmed that BUB1 cDNA restored the growth capacity of FOXM1-silenced HUH7 cells (Fig. 5S, S5D). Meanwhile, overexpression of BUB1 inhibited the apoptosis of HUH7 cells induced by FOXM1 shRNA (Fig. 5T, S5E). Subsequent animal experiments showed that knockdown of BUB1 significantly inhibited the growth of HUH7 xenograft tumors (Fig. 5U-X, S5F). Compared with the NC KD group, the tumor volume and tumor weight in the BUB1 KD group were significantly reduced (Fig. 5U-W, S5G). IHC results indicated that silencing BUB1 expression could significantly inhibit Ki-67 expression, thereby reducing the malignancy of xenograft tumors (Fig. 5X, S5H). These findings indicate that BUB1 is a key downstream target regulated by FOXM1. BUB1 plays a significant role in the malignant progression of HCC, and FOXM1 exerts its oncogenic function by regulating the expression of BUB1.

### BUB1 promotes DNA repair, cell cycle progression, stemness, and migration in HCC cells

**Figure 6:**
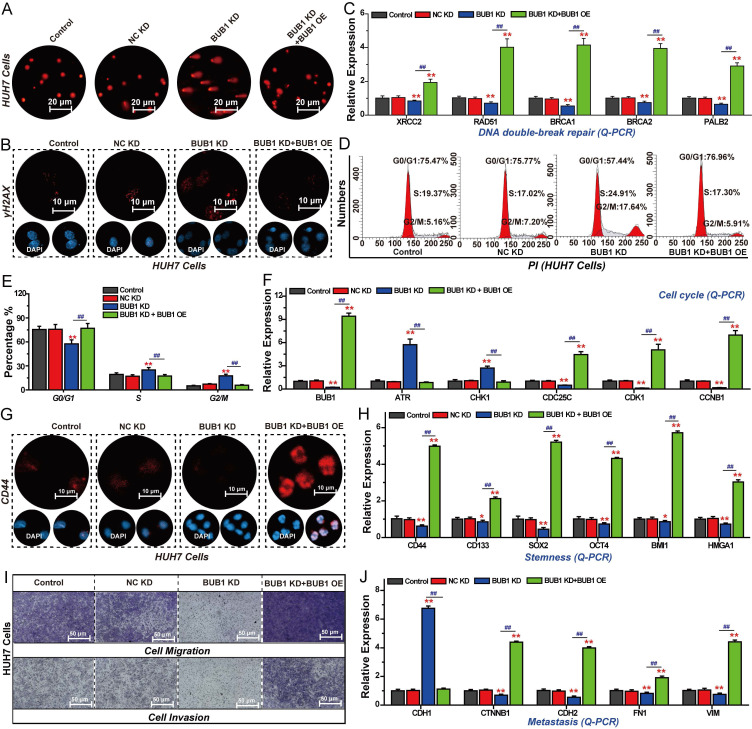
** Inhibition of BUB1 suppresses HCC cell DNA repair, stemness, invasion, and migration. (A)** Effects of BUB1 on DNA damage analyzed by comet assay. **(B)** Effects of BUB1 on γH2AX expression analyzed by IF. **(C)** Effects of BUB1 on the expression of DNA repair-related genes analyzed by Q-PCR. **(D)** Effects of BUB1 on cell cycle progression analyzed by flow cytometry. **(E)** Percentage of cell cycle at G0/G1, S, and G2/M phases. **(F)** Effects of BUB1 on the expression of cell cycle-related genes analyzed by Q-PCR. **(G)** Effects of BUB1 on CD44 expression in HUH7 cells analyzed by IF. **(H)** Effects of BUB1 on stemness-related gene expression in HUH7 cells analyzed by Q-PCR. **(I)** Effects of BUB1 on HUH7 cell invasion and migration. **(J)** Effects of BUB1 on HUH7 cell invasion and migration. Images were randomly selected from five replicates. Data from three independent experiments were statistically analyzed using one-way ANOVA: ^*^P<0.05, ^**^P<0.01 vs. NC KD group, ^#^P < 0.05, ^##^P < 0.01 vs. BUB1 KD group.

As a key downstream target of FOXM1, BUB1 plays crucial role in regulating the malignant progression of HCC. We therefore investigated the effects of BUB1 on HCC cell cycle progression, DNA repair, stemness, invasion, and migration. The results of the comet assay showed that knockdown of BUB1 induced DNA damage in HUH7 cells, and restoring the expression of BUB1 could reverse this effect of promoting DNA damage (Fig. 6A). At the same time, silencing BUB1 expression in HUH7 cells significantly promoted the expression of γH2AX and inhibited the expressions of XRCC2, RAD51, BRCA1, BRCA2, and PALB2 (Fig. 6B, C). Restoring the expression of BUB1 reversed the changes of these genes in FOXM1 silenced group. Subsequent cell cycle experiments demonstrated that silencing the expression of BUB1 could arrest the cell cycle at the S phase and the G2/M phase. Restoring the expression of BUB1 could reverse this change in the cell cycle (Fig. 6D, E). The Q-PCR results showed that BUB1 also regulated the expression of ATR, CHK1, CDC25C, CDK1 and CCNB1, promoting the transition of the cell cycle from the G2 phase to the M phase (Fig. 6F). IF and Q-PCR results showed that silencing BUB1 expression could inhibit the expression of stemness-related targets such as CD44, CD133, SOX2, OCT4, BMI1 and HMGA1, thereby blocking HCC cell stemness (Fig. 6G, H). Invasion and migration assays revealed that suppressing BUB1 expression significantly impaired the invasive and migratory capacities of HUH7 cells (Fig. 6I, S6). Subsequent Q-PCR analysis showed that BUB1 regulates the expression of EMT-related genes CDH1, CDH2, CTNNB1, FN1, and VIM to promote EMT-mediated cell invasion and migration (Fig. 6J). These findings demonstrate that BUB1 drives the malignant progression of pancreatic cancer cells by regulating proliferation, apoptosis, DNA repair, cell cycle progression, stemness, invasion, and migration.

### Inhibition of BUB1 increased the sensitivity of HCC cells to FDI-6

**Figure 7:**
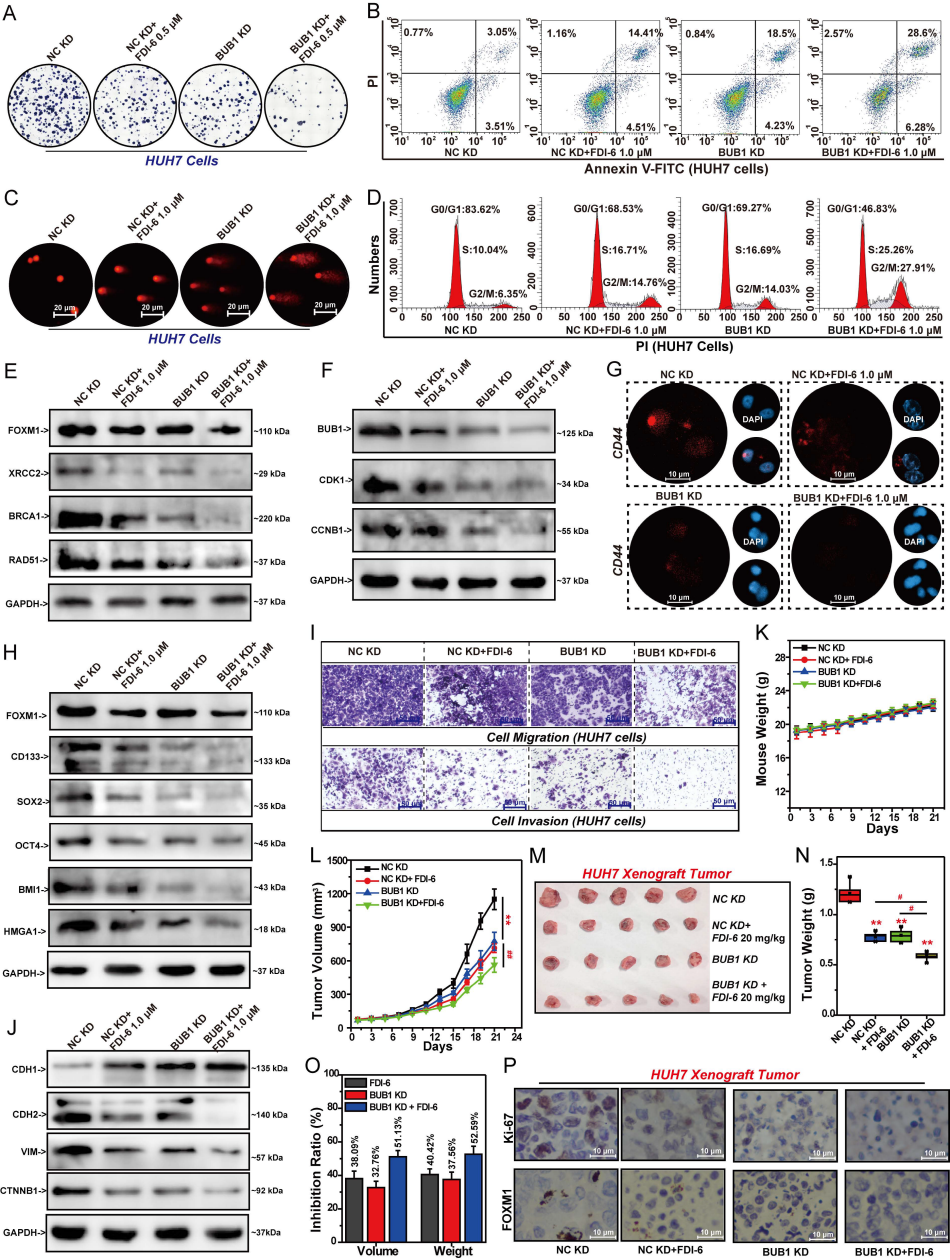
** Knockdown of BUB1 enhances HCC cell sensitivity to FOXM1 inhibitor FDI-6. (A)** Effect of BUB1 shRNA on FDI-6-mediated inhibition of colony formation in HUH7 cells. **(B)** Effect of BUB1 shRNA on FDI-6-induced apoptosis. **(C)** Effect of BUB1 shRNA on FDI-6-induced DNA damage. **(D)** Effect of BUB1 shRNA on the G2/M phase arrest caused by FDI-6. **(E)** Western blot analysis of BUB1 shRNA and FDI-6 effects on DNA repair-related gene expression. **(F)** Western blot analysis of BUB1 shRNA and FDI-6 effects on cell cycle-related gene expression. **(G)** IF analysis of BUB1 shRNA and FDI-6 effects on CD44 expression. **(H)** Western blot analysis of BUB1 shRNA and FDI-6 effects on stemness-related gene expression. **(I)** Effects of BUB1 shRNA on FDI-6 mediated suppression of HCC cell invasion and migration. **(J)** Western blot analysis of BUB1 shRNA and FDI-6 effects on EMT-related gene expression. **(K)** Effects of BUB1 shRNA and FDI-6 on mouse weight. **(L)** Effects of BUB1 shRNA and FDI-6 on tumor volume. **(M)** The photos of HUH7 xenograft tumors in each group. **(N)** Effects of BUB1 shRNA and FDI-6 on tumor weight. **(O)** The inhibition ratios of BUB1 shRNA and FDI-6 on tumor volume and weight. **(P)** IHC analysis of the effects of BUB1 shRNA and FDI-6 on Ki-67 and FOXM1 expression Images were randomly selected from five replicates. Data from three independent experiments were statistically analyzed using one-way ANOVA: ^*^P<0.05, ^**^P<0.01 vs. NC KD group, ^#^P < 0.05, ^##^P < 0.01 vs. BUB1 KD+FDI-6 group.

FOXM1 drives the malignant proliferation of HCC cells by promoting BUB1, highlighting the pivotal role of the FOXM1/BUB1 axis in cancer progression. Therefore, we investigated the effect of BUB1 on the sensitivity of HCC cells to FOXM1 inhibitor FDI-6. Experimental results showed that knockdown of BUB1 significantly enhanced the inhibitory effect of FDI-6 on the formation of HUH7 colonies. Compared with the FDI-6-treated group and BUB1 KD group, the combination of BUB1 shRNA and FDI-6 significantly reduced the number of HUH7 colonies (Fig. 7A, S7A). Similarly, knockdown of BUB1 significantly promoted FDI-6-induced apoptosis in HUH7 cells (Fig. 7B, S7B). Subsequent comet assay results indicated that the knockdown of BUB1 significantly enhanced the promoting effect of FDI-6 on DNA damage (Fig. 7C). The combined use of BUB1 shRNA and FDI-6 significantly caused the cell cycle to arrest at the S phase and G2/M phase (Fig. 7D, S7C). The results of Western blot confirmed that knockdown of BUB1 could significantly enhance the effect of FDI-6 on the expression of cell cycle-related proteins and DNA repair-related proteins, such as CDK1, CCNB1, XRCC2, BRCA1 and Rad51 (Fig. 7E, F, S7D, E). The results of the IF and Western blot experiments indicated that knockdown of BUB1 or FDI-6 could significantly inhibit the expression of stemness-related targets CD44, CD133, SOX2, OCT4, BMI1, and HMGA1. Moreover, knockdown of FOXM1 enhanced the inhibitory effect of FDI-6 on these stemness-related genes (Fig. 7G, H, S7F). Compared with the BUB1 KD group or FDI-6-treated group, the combined treatment group significantly reduced the invasion and migration abilities of HUH7 cells (Fig. 7I, S7G).

Knockdown of BUB1 or FDI-6 significantly suppressed CDH2, VIM, and CTNNB1 expression while promoting CDH1 expression. Silencing BUB1 expression enhanced the inhibitory effect of FDI-6 on pancreatic cancer cell invasion and migration by regulating the expression of these EMT-related genes (Fig. 7J, S7H). Based on the above research results, we established stable BUB1-knockdown HUH7 cells to generate CDX tumors. After 21 days of treatment, knockdown of BUB1 or FDI-6 treatment showed no impact on mouse body weight (Fig. 7K). Knockdown of BUB1 or treatment with 20 mg/kg FDI-6 significantly suppressed both the volume and weight of HUH7 xenograft tumors (Fig. 7L-N). The tumor volume inhibition rate in the combined treatment group increased from 32.76% in the BUB1 KD group and 38.09% in the FDI-6 treatment group to 51.13% (Fig. 7O). IHC analysis revealed that the Ki-67 level in the combined treatment group was significantly lower than that in the BUB1 KD group or the FDI-6 treatment group (Fig. 7P, S7I).

### Targeting FOXM1/BUB1 axis significantly inhibits HCC proliferation

**Figure 8:**
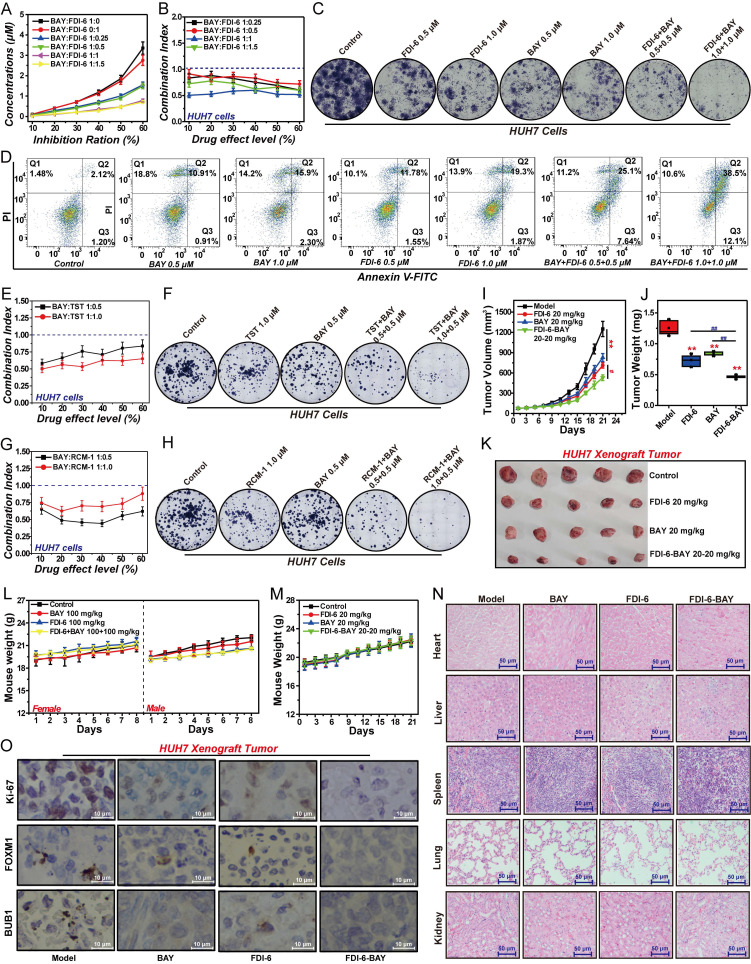
** FOXM1 inhibitors and BAY synergistically inhibit proliferation of HCC cells and tumors. (A)** Effect of BAY and FDI-6 on the proliferation of HUH7 cells. **(B)** The CI values of the combinations of BAY and FDI-6 at different concentration ratios. **(C)** Effect of BAY, FDI-6 and their combination on the colony formation of HUH7 cells. **(D)** Effect of BAY, FDI-6 and their combination on the apoptosis of HUH7 cells. **(E)** The CI values of the combinations of TST and FDI-6 at different concentration ratios. **(F)** Effect of TST, FDI-6 and their combination on the colony formation of HUH7 cells. **(G)** The CI values of the combinations of RCM-1 and FDI-6 at different concentration ratios. **(H)** Effect of RCM-1, FDI-6 and their combination on the colony formation of HUH7 cells. **(I)** Effect of BAY, FDI-6 and their sequential combination on tumor volume. **(J)** Effect of BAY, FDI-6 and their sequential combination on tumor weight. **(K)** The photos of HUH7 xenograft tumors in each group. **(L)** Acute toxicity analysis of 100 mg/kg FDI-6, 100 mg/kg BAY, and their combination in mice. **(M)** Effect of BAY, FDI-6 and their sequential combination on mouse weight. **(N)** Effect of BAY, FDI-6 and their sequential combination on the heart, liver, spleen, lung and kidney in mice. **(O)** Effect of BAY, FDI-6 and their sequential combination on the expression of Ki-67, FOXM1, and BUB1 in HUH7 xenograft tumors. Images were randomly selected from five replicates. Data from three independent experiments were statistically analyzed using one-way ANOVA: ^*^P<0.05, ^**^P<0.01 vs. control group, ^#^P < 0.05, ^##^P < 0.01 vs. BAY-FDI-6 group.

Targeting the FOXM1/BUB1 axis may be an effective strategy for the treatment of HCC. Therefore, we investigated the effects of FOXM1 inhibitor and BUB1 inhibitor on the proliferation of HCC cells and xenograft tumors. We selected the BUB1 inhibitor BAY, and the three inhibitors of FOXM1, namely FDI-6, TST and RCM-1, for subsequent experimental studies. Our results demonstrated that when the concentration ratio of BAY to FDI-6 ranged between 1:0.25 and 1:1.5, the two inhibitors synergistically inhibited the proliferation of HUH7 cells. The optimal synergistic effect was observed at a 1:1 ratio, with the CI value less than 0.7 (Fig. 8A, B). Further colony formation assays confirmed that at a 1:1 ratio of BAY to FDI-6, there was a significant synergistic inhibition of colony formation in HUH7 cells (Fig. 8C, S8A). Apoptosis assay also confirmed that FDI-6 and BAY at 1:1 synergistically promoted the apoptosis of HUH7 cells. After HUH7 cells were treated with the combination of 0.5 μM BAY and 0.5 μM FDI-6, the corresponding apoptosis rate increased from 11.8% in the 0.5 μM BAY-treated group and 13.3% in the 0.5 μM FDI-6-treated group to 32.74%. Similarly, the apoptosis rate of HUH7 cells treated with the combination of 1.0 μM BAY and 1.0 μM FDI-6 was 50.6%, significantly higher than 18.2% in the 1.0 μM BAY treated group and 21.2% in the 1.0 μM FDI-6 treated group (Fig. 8D, S8B). Subsequently, we validated the effects of BAY in combination with TST and RCM-1 on HUH7 cell proliferation. Similar to the previous findings, BAY combined with TST at a concentration ratio of 1:0.5 to 1:1, and BAY combined with RCM-1 at a ratio of 1:0.5 to 1:1, both exhibited synergistic inhibition of HUH7 cell proliferation and colony formation (Fig. 8E-H, S8C-F). Treatment with 20 mg/kg FDI-6 or 20 mg/kg BAY significantly suppressed both the volume and weight of HUH7 xenograft tumors (Fig. 8I-K). Compared with the group treated with 20 mg/kg FDI-6 alone or the group treated with 20 mg/kg BAY alone, the sequential treatment approach of first treating with 20 mg/kg FDI-6 for 10 days and then treating with 20 mg/kg BAY for 10 days significantly inhibited the malignant proliferation of HUH7 xenograft tumors (Fig. 8I-K). The tumor volume inhibition rate in the sequential combined treatment group increased from 33.74% in the BAY treatment group and 43.04% in the FDI-6 treatment group to 57.58% (Fig. S8H). Subsequent acute toxicity experiments showed that 100 mg/kg of BAY, 100 mg/kg of FDI-6, and their combination did not cause the death of mice and had a minor impact on the body weight of mice (Fig. 8L). Meanwhile, after 21 days of treatment, both 20 mg/kg BAY and 20 mg/kg FDI-6 had no effect on the body weight of the mice and the damage to the heart, liver, spleen, lung and kidney tissues (Fig. 8M, N). This suggests that the combination drug strategy targeting the FOXM1/BUB1 axis has high safety and potent antitumor effects. IHC analysis revealed that the Ki-67 level in the sequential combined treatment group was significantly lower than that in the BAY treatment group or the FDI-6 treatment group (Fig. 8O, S8G).

### The FOXM1/BUB1 axis collaboratively drives the malignant progression of HCC

**Figure 9:**
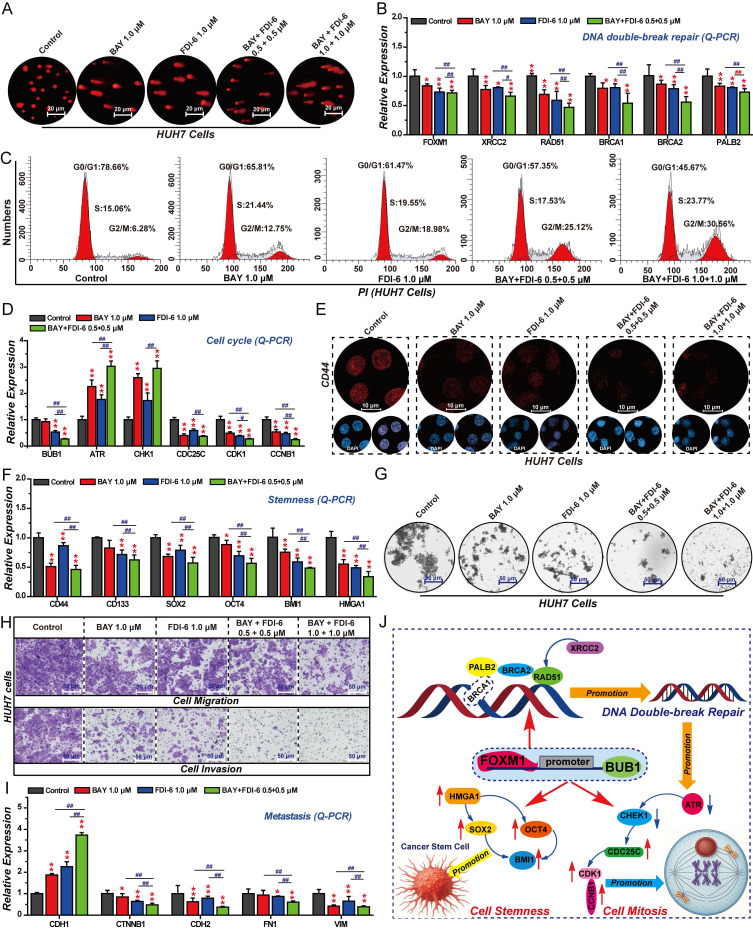
** FOXM1/BUB1 axis drives HCC cell DNA repair, G2/M transition, stemness, migration, and invasion. (A)** Effect of BAY, FDI-6 and their combination on DNA damage in HUH7 cells. **(B)** Effect of BAY, FDI-6 and their combination on DNA repair-related genes in HUH7 cells analyzed by Q-PCR. **(C)** Effect of BAY, FDI-6 and their combination on cell cycle progression in HUH7 cells. **(D)** Effect of BAY, FDI-6 and their combination on cell cycle-related genes in HUH7 cells analyzed by Q-PCR. **(E)** Effect of BAY, FDI-6 and their combination on CD44 expression in HUH7 cells analyzed by IF. **(F)** Q-PCR analysis of the effects of BAY, FDI-6 and their combination on the expression of cell cycle-related genes in HUH7 cells. **(G)** Effect of BAY, FDI-6 and their combination on the formation of HUH7 spheres. **(H)** Effect of BAY, FDI-6 and their combination on the migration and invasion of HUH7 cells. **(I)** Q-PCR analysis of the effects of BAY, FDI-6 and their combination on the expression of EMT-related genes in HUH7 cells. **(J)** The molecular mechanism of the FOXM1/BUB1 axis in regulating HCC malignancy. Images were randomly selected from five replicates. Data from three independent experiments were statistically analyzed using one-way ANOVA: ^*^P<0.05, ^**^P<0.01 vs. control group, ^#^P < 0.05, ^##^P < 0.01 vs. BAY-FDI-6 group.

The FOXM1/BUB1 axis drives the malignant proliferation of HCC cells. Both FOXM1 and BUB1 are involved in regulating DNA repair, cell cycle progression, stemness, invasion and migration of HCC cells. Therefore, we further explored the mechanism of the FOXM1/BUB1 axis in exerting its oncogenic function. Comet assay results indicated that the combination of FDI-6 and BAY could synergistically promote DNA damage in HUH7 cells (Fig. 9A). The Q-PCR results showed that compared with the FDI-6 alone treated group and the BAY alone treated group, the combined treatment of FDI-6 and BAY had a more significant inhibitory effect on the expression of XRCC2, RAD51, BRCA1, BRCA2 and PALB2, suggesting that targeting the FOXM1/BUB1 axis can inhibit the homologous recombination repair pathway for DNA double-strand damage (Fig. 9B).

The results of cell cycle analysis showed that the combination of 1.0 μM FDI-6 and 1.0 μM BAY could synergistically arrest the cell cycle at the G2/M phase (Fig. 9C, S9A). Compared with the 1.0 μM FDI-6 alone treated group and the 1.0 μM BAY alone treated group, the combined treatment of 1.0 μM FDI-6 and 1.0 μM BAY had a more significant regulatory effect on the expression of ATR, CHK1, CDC25C, CDK1, and CCNB1, impairing the transition from the G2 phase to the M phase (Fig. 9D). IF and Q-PCR showed that the combination of 1.0 μM FDI-6 and 1.0 μM BAY could synergistically inhibit the expression of CD44 and CD133, as well as the expression of SOX2, OCT4, BMI1 and HMGA1, which are involved in regulating cell stemness (Fig. 9E, F). Subsequent 3D spheroid formation experiments also confirmed that the combination of 1.0 μM FDI-6 and 1.0 μM BAY significantly inhibited the formation of spheroids, reducing the stemness of HCC cells (Fig. 9G, S9B). Similarly, the combined treatment of 1.0 μM FDI-6 and 1.0 μM BAY was able to synergistically inhibit the migration and invasion of HUH7 cells (Fig. 9H, S9C). Compared with the 1.0 μM FDI-6 alone treated group and the 1.0 μM BAY alone treated group, the combined treatment of 1.0 μM FDI-6 and 1.0 μM BAY had a more significant regulatory effect on the expression of CDH1, CTNNB1, CDH2, FN1 and VIM (Fig. 9I). In summary, the FOXM1/BUB1 axis drives the progression of HCC by promoting cell proliferation, G2/M transition, DNA damage repair, stemness, migration, and invasion.

## Discussion

HCC ranks as the sixth most prevalent malignancy and fourth leading cause of cancer-related mortality worldwide, representing an aggressive hepatocyte-derived cancer with persistently poor prognosis 17-19. Projections indicate a continued rise in HCC mortality over the next decade, underscoring the urgent need for innovative therapeutic strategies to enhance treatment efficacy, reduce recurrence, and prolong survival in advanced-stage patients 17,18. Recent advances in molecular targeted therapies have established mechanism-based combination approaches as a promising option for refractory HCC management 19,20. The oncogenic transcription factor FOXM1 demonstrates consistent overexpression in multiple malignancies, with expression levels positively correlating with aggressive disease progression and poor prognosis 9-12. Substantial evidence implicates FOXM1 in driving critical cancer hallmarks, including proliferative signaling, DNA damage repair, cell cycle progression, stemness maintenance, metastatic dissemination, and therapeutic resistance 9,10. Notably, FOXM1 functionally modulates tumor responsiveness to diverse treatment modalities, including molecular targeted agents, conventional chemotherapeutics, and immune checkpoint inhibitors 9-12. These findings indicate FOXM1 inhibition as a compelling strategy to improve therapeutic efficacy and overcome therapeutic resistance in multiple cancer types. As a crucial transcription factor, FOXM1 exerts its biological functions by transcriptionally regulating its downstream target genes 8,9. Based on our previous findings, we hypothesized that BUB1 may serve as a critical FOXM1 effector mediating its tumor-promoting functions. In this study, we systematically investigated the FOXM1/BUB1 regulatory axis and its mechanistic contributions to HCC pathogenesis through multidimensional analysis of DNA damage repair capacity, cell cycle progression, stemness properties, and migratory potential. Our results demonstrate that FOXM1 directly binds to the BUB1 promoter to transcriptionally activate its expression. Therapeutic targeting of this axis significantly suppressed HCC proliferation, impaired DNA repair, arrested cell cycle progression, attenuated stemness, and inhibited cell migration and invasion. These findings establish the FOXM1/BUB1 axis as a promising multi-mechanistic target for advanced HCC therapy.

As master regulators of oncogenic signaling networks, the transcription factor FOXM1 has been shown to be closely associated with malignant proliferation and poor prognosis in multiple cancers, including HCC, due to its overexpression 8-10, 43. Mechanistic studies demonstrate that FOXM1 primarily promotes cancer cell proliferation, DNA repair, cell cycle progression, stemness, and metastasis by regulating downstream target gene expression 8-10. Notably, FOXM1-driven cancer cell stemness and DNA repair are crucial factors contributing to therapeutic resistance and recurrence in many types of cancers 42, 44. Our analysis of clinical samples from HCC patients demonstrated that FOXM1 expression is significantly higher in tumor tissues compared to adjacent normal tissues. Furthermore, a positive correlation between FOXM1 and Ki-67 expression was observed in the tumor tissues, indicating that elevated FOXM1 levels may drive the malignant progression of HCC 45. Indeed, the results of the bioinformatics analysis also revealed a significant association between FOXM1 overexpression and reduced overall survival in HCC patients.

Experimental regulation of FOXM1 activity through either shRNA-mediated knockdown or pharmacological inhibition (FDI-6) significantly suppressed the proliferation of HCC cells and xenograft tumors. Transcriptomic profiling revealed comprehensive regulatory influence of FOXM1 in multiple oncogenic pathways, including DNA repair, cell cycle, stemness, and EMT. Comet assays demonstrated that genetic or pharmacological suppression of FOXM1 induces DNA damage accumulation and γH2AX expression. H2AX is phosphorylated into γH2AX upon the accumulation of DNA double-strand breaks, indicating the involvement of FOXM1 in regulating DNA double-strand breaks repair 37. Subsequent mechanistic studies revealed that silencing the expression of FOXM1 with FOXM1 shRNA and inhibiting the function of FOXM1 with FDI-6 significantly inhibited the expression of XRCC2, RAD51, BRCA1, BRCA2, and PALB2 in HCC cells. Restoring FOXM1 expression could reverse this inhibitory effect. XRCC2 has been proven to play a crucial role in controlling DNA double-strand break repair 46. It can repair mitochondrial and nuclear DNA damage and promote the malignant behavior in HCC 47. RAD51 is a highly conserved protein that catalyzes DNA repair via homologous recombination pathway 48. The BRCA1-PALB2-BRCA2 axis achieves precise repair of DNA double-strand breaks through the homologous recombination 49. These findings suggest that FOXM1 directly regulates double-strand break repair. FOXM1 promotes G2/M cell cycle progression, thereby facilitating the proliferation of cancer cells. Our study revealed that silencing the expression of FOXM1 or FDI-6 indeed arrested the cell cycle at the G2/M phase, enhanced the expression of ATR and CHK1, and inhibited the expression of CDC25C, CDK1, and CCNB1. Restoring the expression of FOXM1 can reverse this regulatory effect. Activated ATR phosphorylates and activates CHK1, which phosphorylates and inactivates CDC25C to maintain the inactive state of CDK1, thereby inhibiting G2/M progression 39. This indicates that FOXM1 promotes G2/M cell cycle progression by regulating the CDC25C/CDK1/CCNB1 pathway. These findings are consistent with the existing research results on the regulation of the cell cycle process by FOXM1 39-41.

Studies have shown that FOXM1 can promote cell proliferation, invasion and stem cell properties in various types of cancer 10. Our investigation revealed that FOXM1 knockdown significantly suppressed the formation of three-dimensional spheres and concurrently downregulated the expression of stemness-associated factors, including CD44, CD133, SOX2, OCT4, BMI1, and HMGA1. CD44 and CD133 represent well-established surface markers of cancer stem cells, while the transcription factor SOX2 plays a pivotal role in maintaining cancer cell proliferation, stemness properties, and metastatic potential 50. Notably, SOX2 overexpression has been implicated in the development of resistance to both chemotherapy and radiotherapy in various malignancies 51. BMI1 contributes to tumor cell proliferation and stemness maintenance through modulation of multiple oncogenic signaling pathways 52 HMGA1 has been identified as a critical mediator of cancer stemness, invasive capacity, and therapeutic resistance 53. These collective findings demonstrate that FOXM1 promotes HCC stemness through transcriptional activation of key stemness-related genes. Subsequently, we evaluated the effects of FOXM1 on invasion, migration, and EMT-related gene expression in HCC cells. Experimental data demonstrated that FOXM1 inhibition significantly attenuated the invasive and migratory capacities of HCC cells, accompanied by increased expression of CDH1, and decreased expression of CDH2, VIM, CTNNB1 and FN1. These molecular markers are recognized as critical regulators of EMT-driven cancer invasion and metastasis, with the characteristic downregulation of CDH1 and upregulation of VIM, FN1, and CDH2 54. The Wnt/β-catenin signaling pathway regulates tumor stemness and metastasis 55. The comprehensive regulatory roles of FOXM1 in DNA damage repair, cell cycle progression, stemness maintenance, and EMT-mediated invasion and migration collectively establish it as a master regulator of HCC malignant progression. These mechanistic insights strongly support the therapeutic potential of FOXM1-targeted interventions, which may significantly improve both survival outcomes and quality of life for HCC patients through multi-faceted inhibition of tumor progression pathways.

Through integrated analysis of RNA-seq data and PPI networks of FOXM1, we identified BUB1, CDK1, CCNB1 and CTNNB1 are critical downstream effectors mediating the proliferative effects of FOXM1 in HCC. Among the four candidate FOXM1-regulated targets examined, BUB1, CDK1, and CCNB1 demonstrated significant overexpression in HCC cells, with elevated expression levels correlating negatively with patient survival and positively with poor clinical prognosis. BUB1, CDK1, and CCNB1 have been demonstrated to be overexpressed in malignant tumors, leading to cell cycle dysregulation, and closely correlating with tumor metastasis, drug resistance, and poor prognosis 25-28, 37. Studies have confirmed that FOXM1 can transcriptionally activate CCNB1, thereby enhancing cancer cell proliferation 40. Additionally, CDK1 has been shown to phosphorylate FOXM1, which increases its stability and transcriptional activity, further promoting cancer cell proliferation 41.

However, the regulatory relationship between FOXM1 and BUB1 remains unclear. BUB1, a mitotic checkpoint serine/threonine kinase, is a critical component of the cell cycle machinery 25-27. BUB1 plays an essential role in the spindle assembly checkpoint, ensuring accurate chromosome segregation during mitosis 25-27. Dysregulation of BUB1 can result in aneuploidy, a hallmark of cancer progression 25-28. Therefore, we investigated the regulatory relationship between FOXM1 and BUB1. IHC analysis confirmed that BUB1 expression is significantly higher in tumor tissues compared to adjacent normal tissues in HCC patients. Moreover, IHC results indicated that the expression of BUB1 in tumor tissues was positively correlated with the expression of FOXM1, and both of their expressions were positively correlated with the tumor malignancy. This suggests that FOXM1 and BUB1 collaboratively drive tumor malignancy. Further Co-IP experiments revealed that FOXM1 directly interacts with BUB1, suggesting their collaborative role in promoting tumorigenesis. Q-PCR and Western blot analyses demonstrated that FOXM1 upregulates BUB1 expression at both transcriptional and protein levels, whereas BUB1 exhibited no significant effect on FOXM1 expression, thereby establishing a unidirectional regulatory relationship. As a transcription factor, FOXM1 primarily exerts its biological functions by transcriptionally regulating downstream target genes 9. ChIP-qPCR and dual-luciferase reporter assays confirmed that FOXM1 directly binds to the GTAAACC motif located at -293 bp in the BUB1 promoter region, transcriptionally activating BUB1 expression. Functional rescue experiments showed that BUB1 overexpression could partially reverse the anti-proliferative effects of FOXM1 knockdown, providing mechanistic evidence that FOXM1 promotes HCC cell proliferation primarily through BUB1 upregulation. These findings collectively establish BUB1 as a key downstream mediator of FOXM1's oncogenic functions in HCC proliferation, with the FOXM1/BUB1 axis representing a potential therapeutic target for HCC intervention.

As a critical downstream effector of FOXM1 signaling, BUB1 plays a multifaceted role in HCC pathogenesis, significantly promoting the growth of HCC CDX tumors while orchestrating essential cellular processes including DNA damage repair and cell cycle progression 25. Additionally, research also indicates that BUB1 can maintain the stem cell characteristics of cancer cells and enhance the resistance of cancer patients to chemotherapy and radiotherapy 27-30. Our experimental findings demonstrate that genetic or pharmacological inhibition of BUB1 potently suppresses multiple oncogenic processes in HCC, including cell proliferative capacity, DNA repair efficiency, cell cycle progression, stemness maintenance, and invasive and migratory potential, while concomitantly inducing apoptotic cell death. These results establish the FOXM1/BUB1 axis as a master regulator of HCC malignant progression. Systematic therapeutic evaluation revealed that BUB1 knockdown significantly sensitizes both in vitro HCC models and in vivo xenografts to the FOXM1 inhibitor FDI-6. Combination therapy studies further revealed that combination of the BUB1 inhibitor BAY with FOXM1 inhibitor potently suppresses malignant proliferation of HCC cells. Importantly, sequential combination treatment with FDI-6 followed by BAY effectively inhibited HCC xenograft growth *in vivo*, while exhibiting minimal systemic toxicity. Although FDI-6 and BAY are still in the preclinical stage and are associated with significant toxicity and other limitations 13-16. Our experimental results demonstrate that the combined inhibition of FOXM1 and BUB1 represents an effective therapeutic strategy for HCC. Once safer and more potent drug candidates targeting FOXM1 and BUB1 are developed, strategies based on targeting the FOXM1/BUB1 axis hold great potential for clinical translation. Mechanistic studies elucidated that the FOXM1/BUB1 axis drives HCC progression through coordinated regulation of DNA damage repair, G2/M progression, stemness maintenance, and metastatic potential. These findings position dual targeting of the FOXM1/BUB1 axis as a promising therapeutic strategy for advanced and metastatic HCC, offering potential to overcome the limitations of monotargeted therapies.

## Conclusion

In summary, our study provides the first comprehensive characterization of the FOXM1/BUB1 regulatory axis in HCC. FOXM1 binds to the -293 bp region of the BUB1 promoter, specifically at the GTAAACC motif, thereby activating BUB1 transcription. As a critical downstream target of FOXM1, BUB1 promotes malignant progression by enhancing proliferation, G2/M transition, stemness, migration, and invasion of HCC cells. Mechanistically, FOXM1 facilitates tumor progression predominantly through BUB1-mediated pathways. Specifically, the FOXM1/BUB1 axis upregulates key DNA double-strand break repair genes, including BRCA1, RAD51, and XRCC2, thereby promoting efficient DNA damage repair. The FOXM1/BUB1 axis also enhances tumor cell stemness by increasing the expression of pluripotency-associated factors such as OCT4, SOX2, BMI1, and HMGA1. Additionally, this axis drives cell cycle progression and mitosis through activation of the ATR/CDC25C/CDK1/CCNB1 signaling cascade, facilitating HCC cell proliferation (Fig. 9J). Dual inhibition of this axis significantly suppresses tumor growth, impairs DNA repair capacity, blocks the transition from the G2 phase to mitosis, reduces cancer cell stemness, diminishes invasive potential, and concurrently induces apoptosis. Collectively, these findings establish the FOXM1/BUB1 axis as a critical driver of HCC pathogenesis, highlighting its potential as a promising therapeutic target for advanced and metastatic HCC.

## Supplementary Material

Supplementary materials and methods, figures and tables.

## Data Availability

The data will be made available on reasonable request. The raw RNA-seq data have been deposited in the SRA database under accession number PRJNA1272254.

## Abbreviations

FOXM1: forkhead box M1
HCC: hepatocellular carcinoma
BUB1: budding uninhibited by benzimidazoles 1
RNA-seq: RNA sequencing
BAY: BAY-1816032
TST: thiostrepton
LIHC: liver hepatocellular carcinoma
TCGA: the cancer genome atlas
IHC: immunohistochemistry
ROC: receiver operating characteristic
AUC: area under the curve
OS: overall survival
DFS: disease-free survival
KD: knockdown
CDX: cell line-derived xenograft
DEGs: differentially expressed genes
GO: gene ontology
KEGG: kyoto encyclopedia of genes and genomes
PPI: protein-protein interaction
RAD51: radiation defense 51
BRCA1: breast cancer susceptibility gene 1
CDK1: cyclin-dependent kinase 1
CCNB1: cyclin B1
BMI1: B lymphoma Mo-MLV insertion region 1 homolog
HMGA1: high mobility group A1
CD44: cluster of differentiation 44
CTNNB1: β-catenin
VIM: vimentin
CDH1: E-cadherin
EMT: epithelial-mesenchymal transition
γH2AX: phospho-histone H2AX
XRCC2: X-ray repair cross-complementing protein 2
PALB2: partner and localizer of BRCA2
ATR: ataxia telangiectasia and Rad3-related
CHK1: checkpoint kinase 1
CDC25C: cell division cycle 25C
CD133: cluster of differentiation 133, SOX2, SRY-box transcription factor 2
OCT4: octamer-binding transcription factor 4
CDH2: N-cadherin
FN1: fibronectin 1
CI: combination index
Co-IP: co-immunoprecipitation
ChIP-qPCR: chromatin immunoprecipitation Q-PCR
GEPIA: gene expression profiling interactive analysis
HR: log-rank hazard ratio
CCLE: cancer cell line encyclopedia
OE: overexpression
PI: propidium iodide
FBS: fetal bovine serum
BSA: bovine serum albumin
DAPI: diamidino-phenyl-indole
H&E: hematoxylin and eosin
EGF: epidermal growth factor
bFGF: basic fibroblast growth factor
