# Supplementary material for: Mechanistic Insights into the FOXM1/BUB1 axis-Mediated Oncogenic Signaling in Hepatocellular Carcinoma
Source: Int J Biol Sci. 2026 Feb 26;22(6):2986–3012. doi: 10.7150/ijbs.125454 (PMC13050458; doi:10.7150/ijbs.125454)
Supplement: Supplementary file 1 — Supplementary materials and methods, figures and tables. [file ijbsv22p2986s1.pdf]

Supporting information for

Mechanistic Insights into the FOXM1/BUB1 axis-Mediated Oncogenic Signaling in Hepatocellular Carcinoma

Shuping Wang[1,*], Yudong Mao[1,2,*], Tingyu Zeng[1], Tao Yong[1,3], Yu An[1,2], Jipin Li[1,3], Yuan Wang[1], Xiaojun Yang[4], Quanlin Guan[2]

[1] Key Laboratory of Preclinical Study for New Drugs of Gansu Province, Institute of Biochemistry and Molecular Biology, School of Basic Medical Sciences, Lanzhou University, Lanzhou 730000, PR China.

[2] The First School of Clinical Medicine, Lanzhou University, Lanzhou 730000, PR China.

[3] The Second Hospital & Clinical Medical School, Lanzhou University, Lanzhou 730000, China.

[4] Department of Hepatobiliary Surgery, Gansu Provincial Hospital, Lanzhou 730000, China.

[*]The authors contributed equally: Shuping Wang, Yudong Mao

Corresponding author: Shuping Wang, Xiaojun Yang, Quanlin Guan

E-mail: wangsp@lzu.edu.cn (SP. Wang), yangxjmd@aliyun.com (XJ. Yang), guanql@lzu.edu.cn (QL. Guan)

1. Supplementary Materials and Methods

Reagents and antibodies

Anti-BRCA1 rabbit pAb (1:1000, No. 22362-1-AP，RRID:AB_2879090) and CoraLite 594-conjugated Anti-CD44 mouse pAb (1:200, CL594-60224，RRID:AB_2883445) were bought from Proteintech Group (Chicago, IL, USA). Anti-FOXM1 (D3F2B，RRID:AB_2798842) rabbit mAb (1:1000, #20459), and anti-Rad51 (D4B10，RRID:AB_2721109) rabbit mAb (1:1000, #8875) were purchased from Cell Signaling Technology (Boston, MA, USA). Anti-CCNB1 rabbit pAb (1:1000, ab71977，RRID:AB_1310045), Anti-HMGA1 rabbit pAb (1:1000, ab129153，RRID:AB_11139631), Anti-SOX2 rabbit pAb (1:1000, ab92494，RRID:AB_10585428), Anti-BMI1 rabbit pAb (1:1000, ab126783，RRID:AB_11127730), Anti-OCT4 rabbit pAb (1:1000, ab181557，RRID:AB_2687916), Anti-Vimentin rabbit pAb (1:1000, ab16700，RRID:AB_443435), Anti-CD133 rabbit pAb (1:1000, ab222782，RRID:AB_3065213), Anti-E Cadherin mouse pAb (1:1000, ab231303，RRID:AB_2923285), Anti-N Cadherin rabbit pAb (1:1000, ab76011，RRID:AB_1310479), anti-BUB1 rabbit pAb (1:1000,ab195268), anti-CDK1 rabbit pAb (1:1000, ab201008，RRID:AB_2827701), anti-XRCC2 rabbit pAb (1:1000, ab124900，RRID:AB_10971202), and alexa Fluor 647 Anti-gamma H2A.X (phospho S139) rabbit mAb (1:100, ab195189) were obtained from Abcam (Cambridge, England). FDI-6, BAY-1816032, and bovine serum albumin (BSA) were obtained from Sigma-Aldrich (St. Louis, MO, USA). Annexin V-FITC and PI apoptosis-detection kit, MTT cell proliferation assay kit, DAPI, comet assay kit, and crystal violet were bought from KeyGEN Biotech (Nanjing, Jiangsu, China). The stock solution of FDI-6, RCM-1, thiostrepton and BAY-1816032 was prepared by transferring 10 mg to the DMSO at a concentration of 10 mM. Aliquots of the stock solutions were stored at -20 °C. All other chemicals used were analytical grade without purification.

Construction and confirmation of human FOXM1 and BUB1 lentiviral recombination vector

Lentiviral recombination vector of human FOXM1 and BUB1 genes were constructed and purchased from Genechem Co. Ltd. (Shanghai, China). Lentiviral GV341 was used as vector to express human FOXM1 and BUB1 gene, and the restriction sites are BamHI/AgeI. The recombination vector of GV341-cFOXM1 and GV341-cBUB1 were confirmed by PCR and sequencing.

Construction and confirmation of human FOXM1 shRNA and BUB1 shRNA lentiviral recombination vector

Lentiviral recombination vector of the short-hairpin RNA against FOXM1 and the lentiviral recombination vector of the short-hairpin RNA against BUB1 were constructed and purchased from Genechem Co. Ltd. (Shanghai, China). Lentiviral GV112 was used as vector to express FOXM1 shRNA and BUB1 shRNA, and the restriction sites are BamHI and AgeI. The recombination vectors of GV112-shFOXM1 and GV112-shBUB1 were confirmed by sequencing.

2. Supplemental Tables

Table S1. IHC scores of BUB1 and FOXM1 in tumors and adjacent tissues of HCC patients

Gender & Age	Tumor Size (cm^3)	Metastasis	FOXM1		BUB1		Ki67
			Adjacent Tissues	Tumor Tissues	Adjacent Tissues	Tumor Tissues	Tumor Tissues
man（60）	7.6	no	8	134	9	112	230
man（47）	8.0	no	4	91	4	22	44
man（59）	195	no	5	74	11	62	51
man（54）	14	no	1	53	15	34	74
man（78）	42	yes	4	43	1	101	136
man（65）	73.5	no	14	154	4	129	106
Woman（70）	240	no	5	50	12	55	201
Woman（70）	1.5	no	1	131	8	72	120
Woman（56）	9.0	yes	15	123	13	188	181
Woman（51）	37.5	no	9	127	11	94	79
Woman（63）	18	yes	1	59	3	202	58

Table S2. Primer sequences for human genes in Q-PCR.

**Name**	**Sense (5'-3')**	**Antisense (5'-3')**
FOXM1	ACCCAAACCAGCTATGATGCC	TCTCCCGTTTCTGCTCGCAAA
BUB1	GTTTCAGGCTCCTACACTTCC	AGAGATGATCTTATTGACTCCCC
XRCC2	CCTTTTGATTTTGGATAGCCT	GCGATAGTCATTTACAAGCTTC
RAD51	CCCATTTCACGGTTAGAGCA	CTTTGGCTTCACTAATTCCCT
BRCA1	TACTAGGCATAGCACCGTTG	ATGCCTTTGCCAATATTACCTG
BRCA2	TGAAATTAAACGGAAGTTTGC	GAATAAAAGCCCCTAAACCC
PALB2	TCCCAAAAGGCCAAACTCG	GTCATTATCATCAGGCGCAAC
ATR	TGACTCTCAGCCAACCTCC	CAATCCGCAGAAGTCTCGTT
CHK1	TCGATTCTGCTCCTCTAGCTC	CACCACCTGAAGTGACTCGG
CDC25C	CACTTCCTTTACCGTCTGTCC	CTGAGTGGCAGTTATCTCCC
CDK1	ATTTGGAGTATAGGCACCAT	GCCACACTTCATTATTGGGA
CCNB1	TGAGAGCCATCCTAATTGACT	AATTATTCTGCATGAACCGAT
CD44	CACAAATGGCTGGTACGTCT	ATCATCAATGCCTGATCCAGA
CD133	TAGCTACATTATCGACCCCTT	AGTACTTAGCCAGTTTTACCG
SOX2	ATGGCCCAGGAGAACCCCAA	TCGCAGCCGCTTAGCCTCGTC
OCT4	CAGATCAGCCACATCGCCCAG	AGCAGCCTCAAAATCCTCTCGTT

BMI1	TCTTCTTGTTTGCCTAGCC	ATTTACTGATGATTTTCGAGGT
HMGA1	CCAAGCAGGAAAAGGACGGCACT	GGGCTCCTTCTGACTCCCTACCA
CDH1	ACCATTCAGTACAACGACCCAA	GGCCCCTTCACAGTCACAC
CDH2	GAGTTTACTGCCATGACGTT	CTGATTCTGTACACTGCGTTC
CTNNB1	CCACTAATGTCCAGCGTTT	TGGTCCTCGTCATTTAGCAG
VIM	AAATGGCTCGTCACCTTCGT	AAATCCTGCTCTCCTCGCCTT
FN1	GCCACTTCTGTGAACATCCCT	AACTTGGTCCACAGTCGTGTC
GAPDH	GAAACTGTGGCGTGATGGC	CACCACTGACACGTTGGCAG

Table S3. Top50 genes in FOXM1 PPI network.

Protein 1	Protein 2	Co-expression	Combined score
FOXM1	MYBL2	0.523	0.998
FOXM1	CDC25B	0.207	0.918
FOXM1	DLGAP5	0.787	0.872
FOXM1	NCAPG	0.658	0.86
FOXM1	CCNB1	0.683	0.988
FOXM1	TROAP	0.81	0.828
FOXM1	ESPL1	0.829	0.923
FOXM1	KIF11	0.818	0.934
FOXM1	CREBBP	0	0.834
FOXM1	EP300	0.044	0.957
FOXM1	STAT3	0.056	0.893
FOXM1	CDK2	0.217	0.893
FOXM1	BUB1B	0.812	0.944
FOXM1	CCNB2	0.504	0.895
FOXM1	MELK	0.791	0.973
FOXM1	PLK1	0.685	0.995
FOXM1	TPX2	0.788	0.932
FOXM1	LIN37	0	0.86
FOXM1	BIRC5	0.808	0.958
FOXM1	BUB1	0.638	0.825
FOXM1	CDC25A	0.396	0.936
FOXM1	AURKB	0.694	0.889
FOXM1	CDC25C	0.378	0.823
FOXM1	SPAG5	0.785	0.9
FOXM1	LIN9	0.084	0.894
FOXM1	SMAD3	0.051	0.911
FOXM1	LIN54	0.049	0.876
FOXM1	E2F1	0.337	0.822
FOXM1	RRM2	0.681	0.835
FOXM1	MCM10	0.642	0.844
FOXM1	PBK	0.548	0.845

FOXM1	DEPDC1	0.522	0.845
FOXM1	ASPM	0.668	0.848
FOXM1	CEP55	0.704	0.86
FOXM1	PRC1	0.805	0.87
FOXM1	CDCA3	0.829	0.885
FOXM1	KIF2C	0.825	0.888
FOXM1	ALKBH5	0	0.893
FOXM1	UBE2C	0.663	0.894
FOXM1	KIF4A	0.835	0.897
FOXM1	MKI67	0.836	0.91
FOXM1	HJURP	0.815	0.918
FOXM1	TOP2A	0.665	0.927
FOXM1	CDCA8	0.819	0.93
FOXM1	CDC20	0.783	0.937
FOXM1	KIF20A	0.802	0.944
FOXM1	CCNA2	0.725	0.985
FOXM1	CENPF	0.747	0.989
FOXM1	CDK1	0.661	0.991
FOXM1	CTNNB1	0	0.994

Table S4. Top50 genes in BUB1 PPI network.

Protein 1	Protein 2	Co-expression	Combined score
BUB1	AURKA	0.836	0.971
BUB1	MAD2L2	0.109	0.968
BUB1	NCAPH	0.838	0.933
BUB1	DLGAP5	0.949	0.986
BUB1	NCAPG	0.908	0.976
BUB1	CCNB1	0.846	0.992
BUB1	ESPL1	0.775	0.99
BUB1	KIF23	0.855	0.958
BUB1	KIF11	0.912	0.985
BUB1	NDC80	0.932	0.998
BUB1	SGO1	0.724	0.982
BUB1	CENPE	0.839	0.991
BUB1	NUF2	0.901	0.994
BUB1	SPC25	0.814	0.967
BUB1	BUB1B	0.92	0.999
BUB1	CCNB2	0.892	0.998
BUB1	MAD2L1	0.841	0.999
BUB1	MELK	0.936	0.984
BUB1	PLK1	0.587	0.996
BUB1	TPX2	0.847	0.958

BUB1	BIRC5	0.844	0.979
BUB1	CDC23	0.097	0.934
BUB1	SPAG5	0.886	0.934
BUB1	PBK	0.869	0.935
BUB1	NUSAP1	0.858	0.937
BUB1	HASPIN	0.38	0.938
BUB1	RAE1	0.078	0.939
BUB1	DSN1	0.265	0.944
BUB1	CHEK1	0.794	0.946
BUB1	ECT2	0.904	0.947
BUB1	KIF4A	0.823	0.952
BUB1	UBE2C	0.887	0.954
BUB1	PTTG1	0.744	0.955
BUB1	KIF20A	0.863	0.959
BUB1	MKI67	0.899	0.963
BUB1	CENPA	0.815	0.969
BUB1	CDCA8	0.847	0.971
BUB1	ASPM	0.907	0.976
BUB1	CCNA2	0.879	0.981
BUB1	TOP2A	0.918	0.982
BUB1	CENPF	0.871	0.987
BUB1	ZWINT	0.64	0.991
BUB1	KIF2C	0.862	0.994
BUB1	AURKB	0.827	0.997
BUB1	MAD1L1	0	0.998
BUB1	CDK1	0.89	0.998
BUB1	TTK	0.989	0.999
BUB1	CDC20	0.907	0.999
BUB1	KNL1	0.458	0.999
BUB1	BUB3	0.161	0.999

3. Supplemental Figures

Figure. S1

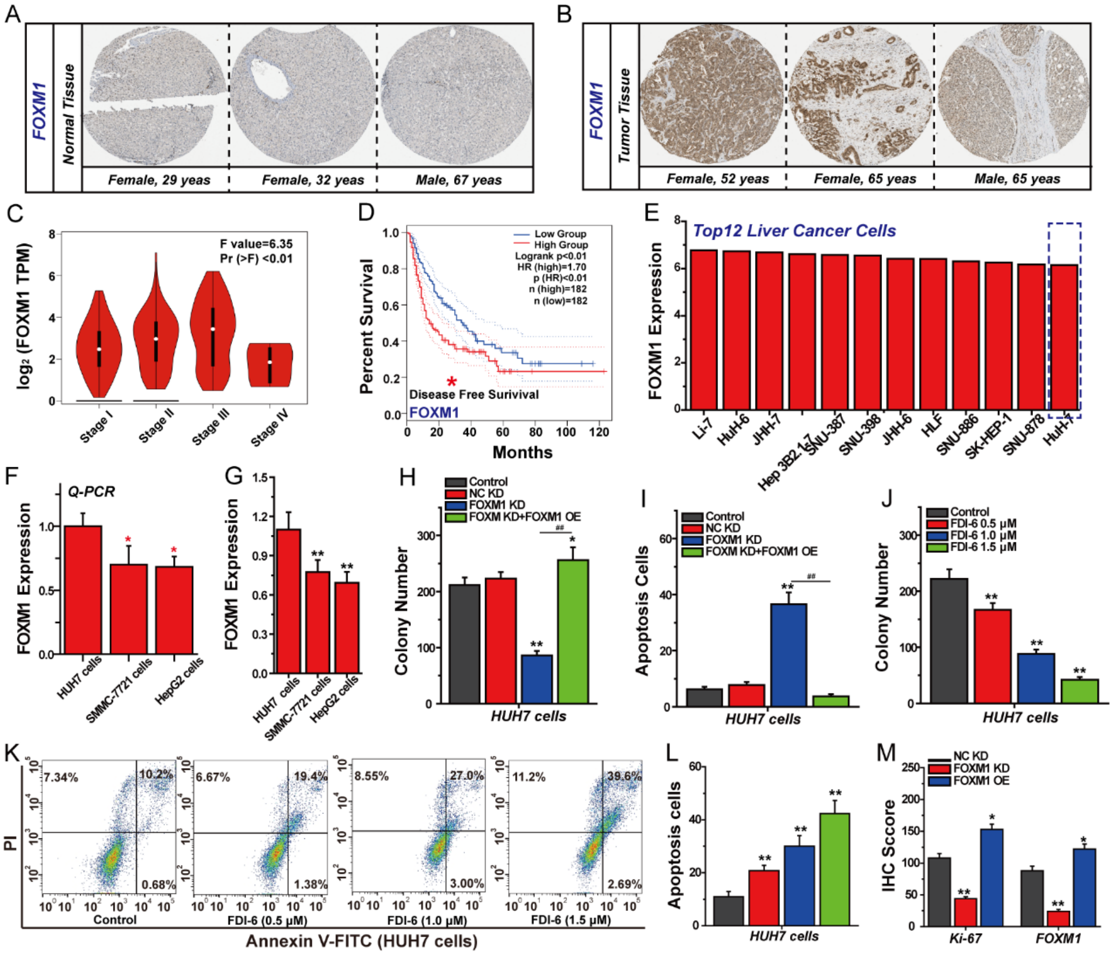

Figure. S1. FOXM1 promotes the proliferation of hepatoma carcinoma cells *in vitro*. (A). The expression of FOXM1 in normal tissues of liver in the human protein atlas. (B). The expression of FOXM1 in HCC tissues in the human protein atlas. (C). FOXM1 expression in different stages of liver hepatocellular carcinoma (LIHC) for TCGA. (D). Correlation between FOXM1 expression levels and survival of HCC patients. (E). Distribution of FOXM1 mRNA expression in different hepatocellular carcinoma cell lines. (F). Relative expression of FOXM1 in HUH7, HepG2, and SMMC-7721 cells analyzed by Q-PCR. (G). Relative expression of FOXM1 in HUH7, HepG2, and SMMC-7721 cells analyzed by Western blots. (H). Number of HUH7 colonies in each group. (I). Percentage of PHUH7 apoptosis cells. (J). Effects of FDI-6 on HUH7 colony numbers. (K). Effects of FDI-6 on the apoptosis of HUH7 cells. (L). Percentage of apoptosis cells. (M). IHC scores of FOXM1 and Ki-67 in HUH7 xenograft tumors. The results from three independent experiments were statistically analyzed using one way ANOVA: *P<0.05, **P<0.01 compared with control group, $^{\#}$P<0.05, $^{\#\#}$P<0.01 compared with FOXM1 KD group.

Figure. S2

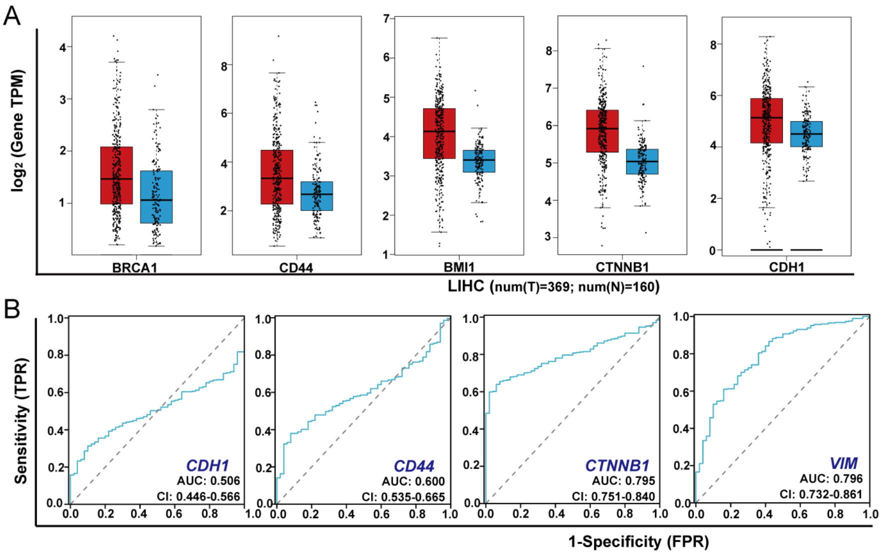

Figure. S2. Prognostic analysis of genes related to cell stemness and EMT. (A). Differential expression of genes between cancer tissues and adjacent tissues of hepatocellular carcinoma in TCGA project. (B). ROC curves for the relationship of gene expression and the survival of patient with LIHC.

Figure. S3

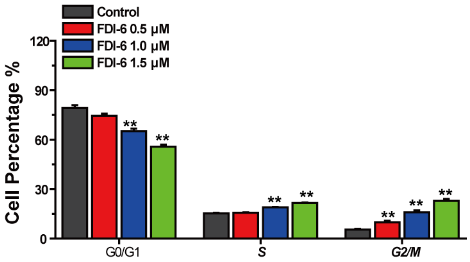

Figure. S3. Effects of FDI-6 on cell cycle progression in hepatocellular carcinoma cells. The results from three independent experiments were statistically analyzed using one way ANOVA: *P<0.05, **P<0.01 compared with control group.

Figure. S4

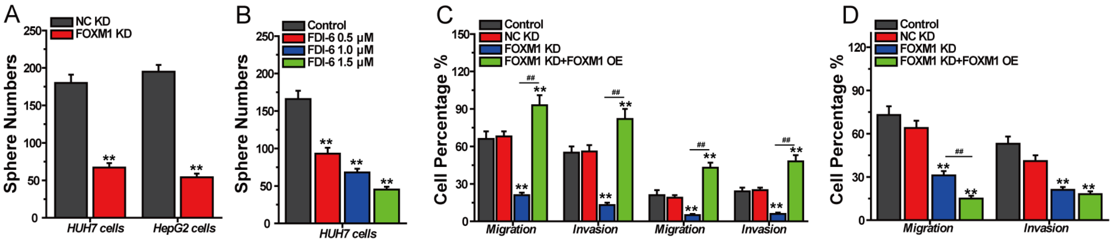

Figure. S4. The effects of FOXM1 on cell stemness, migration and invasion in hepatocellular carcinoma cells. (A). Effects of FOXM1 shRNA on sphere formation of HUH7 and HepG2 cells. (B). Effects of FDI-6 on sphere formation of HUH7 cells. (C). Effects of FOXM1 shRNA on migration and invasion of HUH7 and HepG2 cells. (D). Effects of FDI-6 on migration and invasion of HUH7 cells. The results from three independent experiments were statistically analyzed using one way ANOVA: $^{*}P<0.05$, $^{**}P<0.01$ compared with control group or NC KD group, $^{\#}P<0.05$, $^{\#\#}P<0.01$ compared with FOXM1 KD group.

Figure. S5

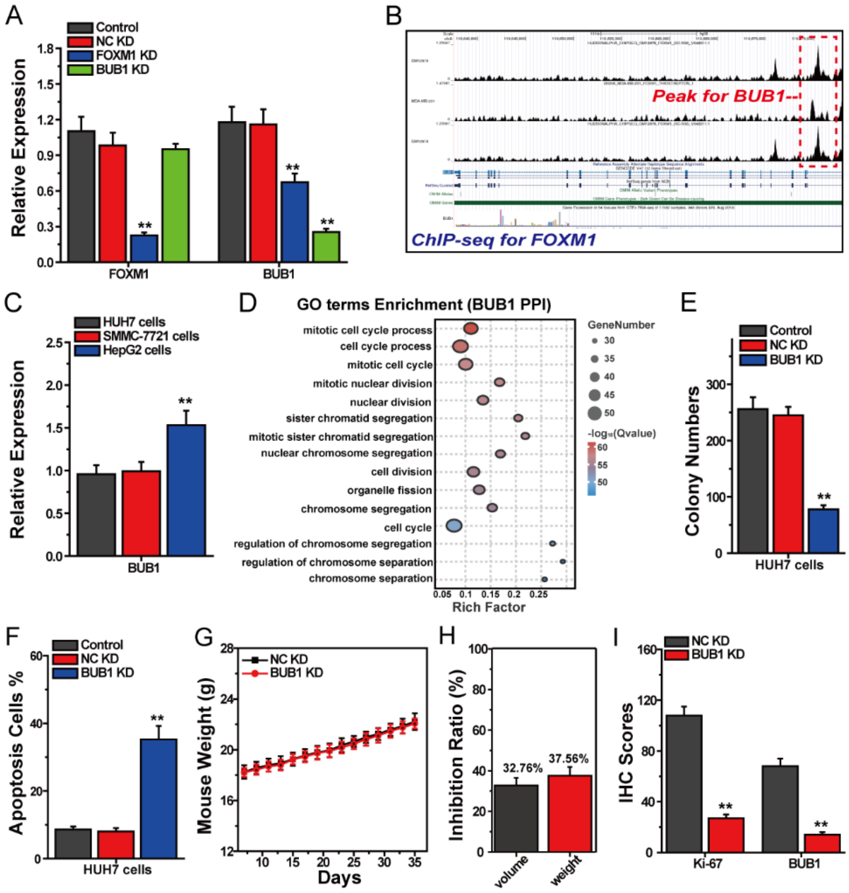

Figure. S5. Effects of BUB1 on the proliferation of hepatocellular carcinoma cells. (A). Relative expression of FOXM1 and BUB1 in HUH7 cells analyzed by Western blot. (B). Binding of FOXM1 to the BUB1 promoter region in ChIP-seq data from Cistrome Data Broweser. (C). Relative expression of BUB1 in different hepatocellular carcinoma cells analyzed by Western blot. (D). CO terms Enrichment analysis of genes in BUB1 PPI network. (E). Colony numbers in each group. (F). Apoptosis cells in each group. (G). Mouse weight in each group. (H). Inhibition ratio of tumor volume and weight in each group. (I). IHC scores of Ki-67 and BUB1 in each group. The results from three independent experiments were statistically analyzed using one way ANOVA: $^{*}P<0.05$, $^{**}P<0.01$ compared with NC KD group or HUH7 cells.

Figure. S6

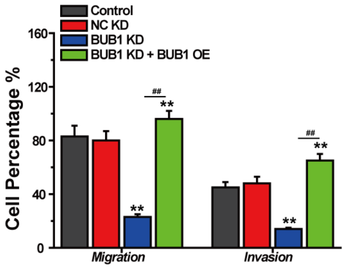

Figure. S6. Effect of BUB1 on the migration and invasion in hepatocellular carcinoma cells. The results from three independent experiments were statistically analyzed using one way ANOVA: $^{*}P<0.05$, $^{**}P<0.01$ compared with NC KD group, $^{\#}P<0.05$, $^{\#\#}P<0.01$ compared with BUB1 KD group.

Figure. S7

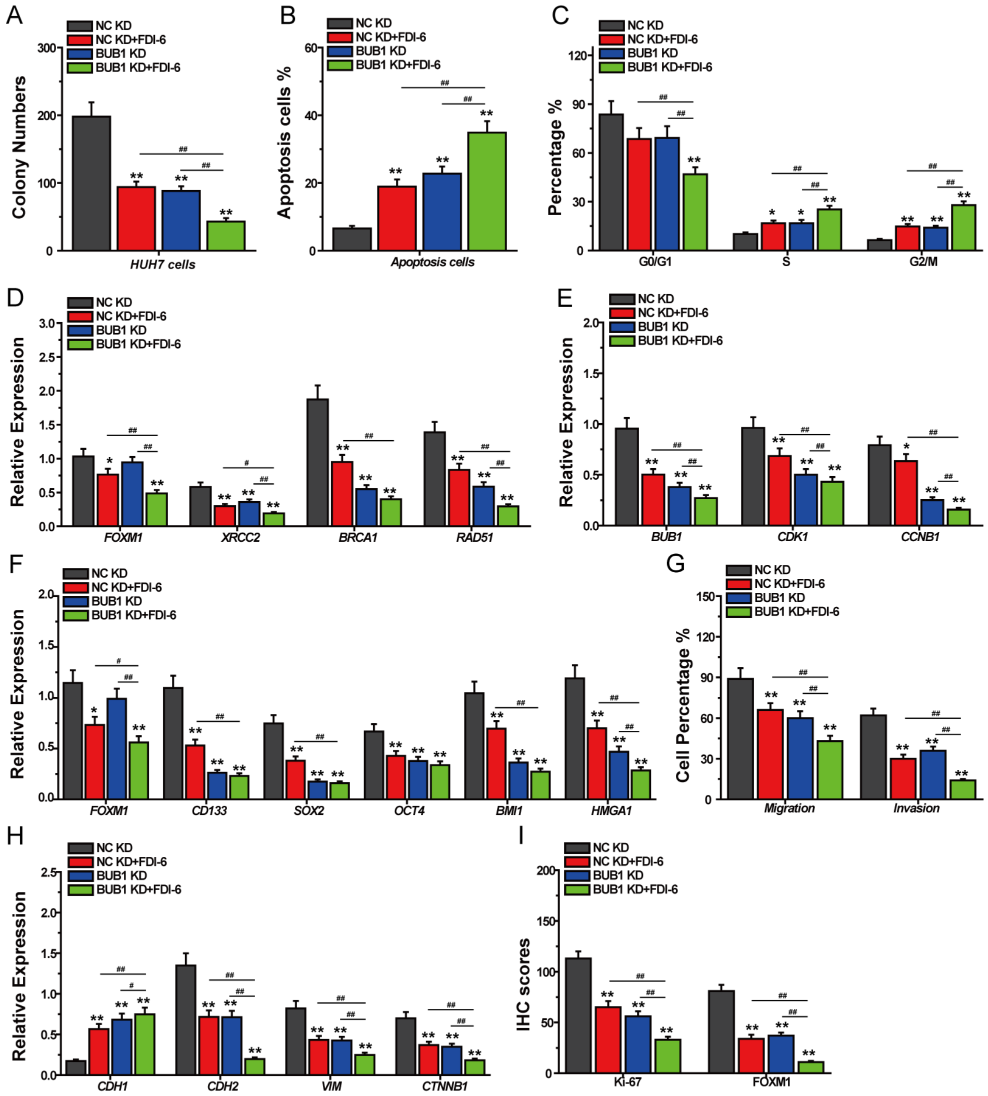

Figure. S7. Effect of BUB1 shRNA and FDI-6 on DNA repair, cell stemness, invasion and migration in hepatocellular carcinoma cells. (A). Colony numbers in HUH7 cells. (B). Percentage of apoptosis cells in each group. (C). Cell cycle progressions in each group. (D). Relative expression of DNA repair-related genes analyzed by Western blot. (E). Relative expression of cell cycle-related genes analyzed by Western blot. (F). Relative expression of stemness-related genes analyzed by Western blot. (G). Percentage of migrated cells and invaded cells in HUH7 cells. (H). Relative expression of EMT-related genes analyzed by Western blot. (I). IHC scores of Ki-67 and FOXM1 in each group. The results from three independent experiments were statistically analyzed using one way ANOVA: $^{*}P<0.05$, $^{**}P<0.01$ compared with NC KD group, $^{\#}P<0.05$, $^{\#\#}P<0.01$ compared with BUB1 KD+FDI-6 group.

Figure. S8

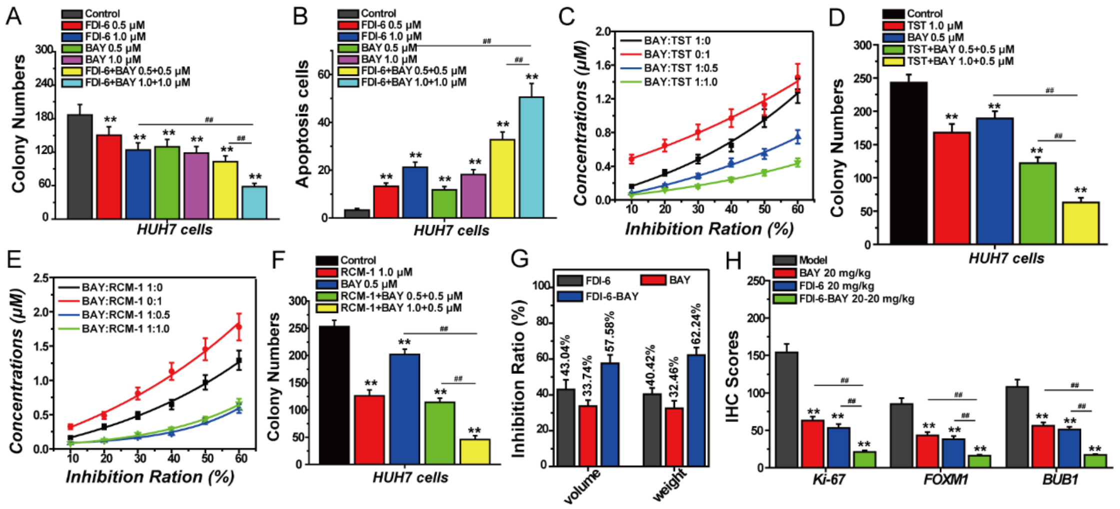

Figure. S8. Effect of FOXM1 inhibitors and the BUB1 inhibitor BAY on the proliferation of hepatocellular carcinoma cells. (A). Colony numbers in each group. (B). Percentage of apoptosis cells in each group. (C). Effect of BAY and TST on the proliferation of HUH7 cells. (D). Effect of BAY and TST on colony formation of HUH7 cells. (E). Effect of BAY and RCM-1 on the proliferation of HUH7 cells. (F). Effect of BAY and RCM-1 on colony formation of HUH7 cells. (G). The inhibition ratios of BAY, FDI-6 and their sequential combination on tumor volume and weight. (H). IHC scores of Ki-67, FOXM1 and BUB1 in each group. The results from three independent experiments were statistically analyzed using one way ANOVA: $^{*}P<0.05$, $^{**}P<0.01$ compared with control group, $^{\#}P<0.05$, $^{\#\#}P<0.01$ compared with FDI-6+BAY group.

Figure. S9

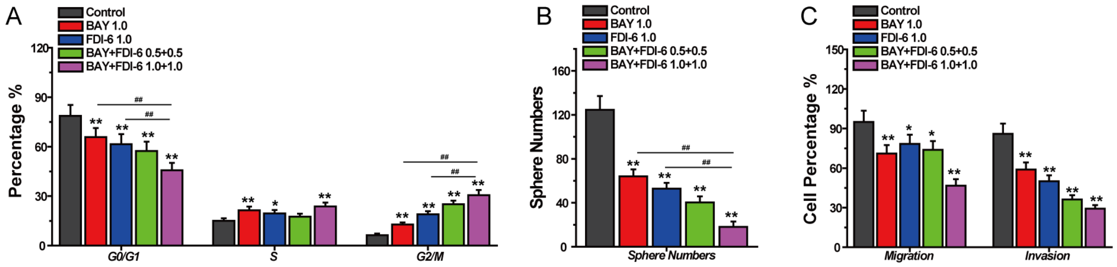

Figure. S9. Effect of FDI-6 and BAY on cell cycle progression, cell stemness, invasion and migration in hepatocellular carcinoma cells. (A). cell cycle progression in each group. (B). Sphere numbers in each group. (C). Percentage of migrated cells and invaded cells in HUH7 cells. The results from three independent experiments were statistically analyzed using one way ANOVA: *P<0.05, **P<0.01 compared with control group, #P<0.05, ##P<0.01 compared with FDI-6+BAY group.
